# Modular response analysis reformulated as a multilinear regression problem

**DOI:** 10.1093/bioinformatics/btad166

**Published:** 2023-04-06

**Authors:** Jean-Pierre Borg, Jacques Colinge, Patrice Ravel

**Affiliations:** Institut de Recherche en Cancérologie de Montpellier, Inserm U1194, Montpellier 34298, France; Institut régional du Cancer Montpellier, Montpellier 34298, France; Université de Montpellier, Montpellier 34090, France; Institut de Recherche en Cancérologie de Montpellier, Inserm U1194, Montpellier 34298, France; Institut régional du Cancer Montpellier, Montpellier 34298, France; Université de Montpellier, Montpellier 34090, France; Faculté de Médecine, Montpellier 34090, France; Institut de Recherche en Cancérologie de Montpellier, Inserm U1194, Montpellier 34298, France; Institut régional du Cancer Montpellier, Montpellier 34298, France; Université de Montpellier, Montpellier 34090, France; Faculté de Pharmacie, Montpellier 34090, France

## Abstract

**Motivation:**

Modular response analysis (MRA) is a well-established method to infer biological networks from perturbation data. Classically, MRA requires the solution of a linear system, and results are sensitive to noise in the data and perturbation intensities. Due to noise propagation, applications to networks of 10 nodes or more are difficult.

**Results:**

We propose a new formulation of MRA as a multilinear regression problem. This enables to integrate all the replicates and potential additional perturbations in a larger, over-determined, and more stable system of equations. More relevant confidence intervals on network parameters can be obtained, and we show competitive performance for networks of size up to 1000. Prior knowledge integration in the form of known null edges further improves these results.

**Availability and implementation:**

The R code used to obtain the presented results is available from GitHub: https://github.com/J-P-Borg/BioInformatics

## 1 Introduction

Biological systems are orchestrated by a multitude of interaction networks. At a cellular scale, the knowledge of protein physical interaction networks, phosphorylation cascades, or gene regulatory networks is central in our understanding of homeostasis, disease, reaction to environmental changes, and many other situations. The inference of biological networks from experimental data has thus attracted much attention from the computational biology community (Huynh-Thu and Sanguinetti 2019).

A common experimental design to learn about the connectivity between a set of molecules of interest is to measure their activity under different conditions. Molecules can be for instance genes, transcripts, proteins, or metabolites, and the notion of activity might relate to their abundance or state, e.g. the phosphorylation level of a protein. Modular response analysis (MRA) is a versatile framework allowing to infer connectivity between molecules (or groups of molecules called modules) $X_{1},\ldots ,X_{N}$ simply assuming that they are related by an equation of the form $dX/dt=f\left(X\right)$, $X={\left(X_{1},\ldots ,X_{N}\right)}^{T}$, and a particular experimental design. This design requires that measures of $X_{i}$ activities are obtained under the systematic perturbation of each molecule activity, and the system finds itself in a steady state for each of those perturbations. The elegance of MRA is that the steady-state assumption enables applying the implicit function theorem leading to a first-order approximation of f(X) without knowing this function explicitly (Kholodenko et al. 2002). Except for the steady-state hypothesis, which is not fulfilled by every system obviously, MRA provides a generic solution involving linear algebra only to explore biological networks quantitatively. It has been applied and extended by a number of researchers (Dorel et al. 2018; Santra et al. 2018; Jimenez-Dominguez et al. 2021; Mekedem et al. 2022). However, MRA is sensitive to measurement noise and to the intensity of the perturbations exerted on network nodes (Andrec et al. 2005; Thomaseth et al. 2018). Different approaches have been suggested to alleviate these problems (Santra et al. 2018), e.g. to perform a bootstrap followed by an estimation of confidence intervals (CIs) of the connectivity coefficients (Santos et al. 2007; Jimenez-Dominguez et al. 2021).

In this report, we combine MRA with multilinear regression. Indeed, MRA equations can be interpreted as a linear regression problem (see below) thus enabling the use of any linear regression strategy. This new formulation hence defines a family of methods, which we will illustrate employing classical algorithms such as the least square, LASSO, or STEP. One advantage of the regression approach is to integrate the treatment of the experimental noise with the model accuracy estimation in terms of residual variance. That is, how well the linear MRA model approximates the potentially nonlinear biological system and how accurate are the estimated network parameters. Using data from DREAM 4 Challenge, we show that we can apply MRA modeling to 10- to 100-node networks with competitive performance, while a standard resolution of MRA equations already encounters difficulties with 10-node networks. Using realistic synthetic networks of sizes up to 1000 nodes (Carré et al. 2017) and varying noise in the data, we confirm those results. We further compare MRA and our multilinear regression variants against general-purpose network inference algorithms such as ARACNE (Margolin et al. 2006), CLR (Faith et al. 2007), and MRNET (Meyer et al. 2007), which we largely outperform.

## 2 Materials and methods

### 2.1 Fundamental definitions

We consider a putative biological network comprised of N modules that potentially interact. A module can be a molecule (transcript, protein, …) or it can represent the contribution to the network of a set of molecules such as a pathway or part of a pathway. In the latter case, one measurement reports for the activity of the whole module. MRA aims at determining signed connectivity coefficients between the modules. The module activity levels are represented as a time-dependent function $X:R^{+}\mapsto R^{N}$. In case the modules were gene transcripts, X(t) could for instance represent their abundance over time. We assume the existence of a continuously differentiable function $f:R^{N}{\times R}^{M}\mapsto R^{N}$ and a vector of intrinsic parameters $P\in R^{M}$, at least one *per* module (hence M≥N), such that


(1) dXdt=fX,P.

The function f is usually unknown. $P_{0}$ represents the biological system in its basal state, i.e. in the absence of any perturbation. We suppose the existence of a time $t_{0}$ after which the system has reached a steady state (including all the perturbed states):


(2) dXdt=0, ∀t≥t0.

Let $X=X(P_{0})$ be a solution of the system (2). The application of the implicit function theorem gives us an exact formula for the connectivity coefficient representing the influence of a module j on another module i (Kholodenko et al. 2002; Jimenez-Dominguez et al. 2021):


(3) $$r_{ij}^{e}=-\left(\frac{X_{j}\frac{\partial f_{i}}{\partial X_{j}}\;}{X_{i}\frac{\partial f_{i}}{\partial X_{i}}}\right)=\;\frac{X_{j}}{X_{i}}\left(\frac{\partial X_{i}}{\partial X_{j}}\right),\;1\leq i\neq j\leq N$$

Note that $r_{ii}^{e}\;$is not defined by Equation (3). By convention, we set $r_{ii}^{e}=-1$, see Jimenez-Dominguez et al. (2021) for an algebraic justification.

Taylor’s development at the first order enables us to write an expression for the system under perturbation ΔP. for 1≤i≤N and with $o\left(h\right)=h\varepsilon (h)$, $\underset{h\to 0}{\lim}\varepsilon (h)=0$, ε(h) continuous in the vicinity of 0.


(4) XiP0+ΔP-XiP0Xi=+∑j=1, j≠iNXjXi∂Xi∂Xj XjP0+ΔP-XjP0Xj+1Xi∑j=1 N∂Xi∂PjΔPj+oΔP,

#### 2.1.1 Standard MRA formulation

Only one perturbation *per* module is considered implying M=N. Experimental measurements obtained under perturbation *q_i_* result from the modification of the intrinsic parameter $P_{i\;}$ that directly affects node i only. Accordingly,


(5) $$\frac{\partial X_{i}}{\partial P_{j}}\neq 0\;\;\mathrm{if}\;\;i=j\;\mathrm{and}\;\frac{\partial X_{i}}{\partial P_{j}}=0\;\mathrm{if}\;i\neq j.$$

We introduce the notation


(6) $${\left(\frac{{\Delta X}_{i}}{X_{i}}\right)}_{q_{k}}=2*\frac{X_{i}\left(P_{0}+{\Delta P}_{k}\right)-X_{i}\left(P_{0}\right)}{X_{i}\left(P_{0}+\Delta P_{k}\right)+X_{i}\left(P_{0}\right)}.$$

Using the mid-point in the denominator of Equation (6) right-term instead of $X_{i}\left(P_{0}\right)$ is customary to avoid divisions by 0. Thanks to Equations (4) and (5), we obtain the system of equations with 1≤i≤N and k≠i:


(7) ΔXiXiqk=∑j=1, j≠iNrijeΔXjXjqk+ oΔP.

Ignoring the error term $o\left(\left\|\Delta P\right\|\right)\;$in (7) that represents departure from linearity, we obtain its linear approximation that is solved by MRA (note that $r_{ij}$ replaces the exact $r_{ij}^{e}$). In particular, writing matrices $r=(r_{ij})$ and $R\;=\left({\left(\frac{{\Delta X}_{i}}{X_{i}}\right)}_{q_{k}}\right)$, the connectivity coefficients are obtained by: an N×N system (Kholodenko et al. 2002).


(8) ΔXiXiqk=∑j=1, j≠iNrijΔXjXjqk

(9) $$r=-{\left[\text{diag}\left(R^{-1}\right)\right]}^{-1}\;R^{-1}$$

### 2.2 A multilinear regression formulation

Equation (9) does not take into account the existence of replicates in the data. It requires a single value for each perturbed state and each module. In practice, *ad hoc* procedures must be added to exploit replicates properly and estimate CIs for the $r_{ij}$ computed after Equation (9). Typically, bootstrap approaches are used that apply Equation (9) to resampled data or Equation (9) is applied to averaged data perturbed by an appropriate noise function (Andrec et al. 2005; Santos et al. 2007; Thomaseth et al. 2018; Jimenez-Dominguez et al. 2021).

An alternative approach is to consider Equation (8) as the homogeneous multilinear regression of $\left(\frac{{\Delta X}_{i}}{X_{i}}\right)$ with regression parameters $r_{ij}$. In that case, the system to solve is not limited to N-1 equations. We can have one equation *per* replicate with any number of replicates. It is even possible to integrate multiple perturbations at each node, e.g. at different intensities or by different means, and hence M≥N. The number of perturbations is also no longer required to be identical at each node. Obviously, we have N such regressions to perform (N-1 variables each) and the regression approach let us estimate $r_{ij}$ variability, directly taking advantage of all the tools provided by the regression literature (Hastie et al. 2009).

### 2.3 Considered regression methods

To solve Equation (8) with a regression approach leaves the choice of the specific regression method free. Here, we considered several standard methods. An obvious choice was least square estimation (LSE) applied to multilinear regression. Because most biological networks are rather sparse and they can reach a certain size, we primarily considered methods able to eliminate the least significant regression parameters. Accordingly, we defined the LSE_CI method as the application of LSE followed by an elimination of all the $r_{ij}$ whose 95% CI included 0.

A second method was threshold linear regression (TLR), which also starts with LSE but eliminates coefficients differently. The rule is to compute a threshold $T_{h}$ as the 25th percentile of all the estimated values $\left|r_{ij}\right|$ and to set to 0 those $r_{ij}$ that are below threshold in absolute value.

The third method was LASSO, a shrinkage estimator. Using LASSO for a node i (the *i*th of the N systems of size N-1 to solve) is defined by:


(10) rijLasso=argminri.⁡∑k=1,KΔXiXiqk-∑j=1, j≠iNrijΔXjXjqk2+ λi ∑j=1, j≠iNrij

The choice of the hyper-parameter $\lambda_{i}$ is determined by cross-validation (CV) (Hastie et al. 2009), and it is hence necessary to have experimental replicates or multiple perturbations at certain nodes.

The fourth option was methods integrating a variable selection scheme. Namely, we used STEP forward (STEP-Fo), STEP backward (STEP-Ba), and Stepwise regression (STEP-Bo) combining both backward and forward (Hastie et al. 2009). They essentially consist in finding subsets of variables minimizing the sum of the residual squares while ignoring the other variables according to different selection strategies. This selection process introduces a bias in the solution, but here, this bias should remain modest because the networks are sparse (roughly 80% of null edges).

### 2.4 Network inference methods

We considered ARACNE (Margolin et al. 2006), CLR (Faith et al. 2007), and MRNET (Meyer et al. 2007). We used their implementation in the Bioconductor R package minet (Meyer et al. 2008).

#### 2.4.1 Selection and discretization of connectivity coefficients

Standard MRA and the regression-based variants we propose all estimate real-valued connectivity coefficients $r_{i,j}$. Methods such as LASSO, LSE_CI, TLR, and STEP-xx include a mechanism to identify coefficients deemed to be significant, the other ones being set to 0. MRA usually outputs coefficients $r_{i,j}$ that are all or almost all nonzero. We applied two techniques to assess standard MRA connectivity coefficient significance. For very small networks (the 6-kinase example), we used a bootstrap (100 repetitions) to estimate a CI on each $r_{i,j}$ and set $r_{i,j}$ to 0 as soon as the CI included 0. For larger networks, this procedure was too slow and we applied a heuristics: we defined a threshold T equal to the top x% of the $r_{i,j}$ in absolute value, and set each $r_{i,j}$ to 0 as soon as $\left|r_{i,j}\right|<T$. The choice of x was either variable to obtain ROC curves or set to a chosen value specified in Section 3.

In cases where it was necessary to compare with a reference network where only the existence and sign of edges were known, we had to discretize the nonzero connectivity coefficient produced by each method to {−1; 0; 1}. To compare performance with network inference algorithms such as ARACNE, CLR, and MRNET, we also had to discretize the connectivity coefficients of some reference networks. We applied the following rules. We denote Solution the matrix of exact coefficients $r_{ij}^{e}$ of the reference network, and MatrCc the matrix of approximated $r_{ij}$ after selection of significant coefficient above. For each row (iRow) and column (iCol) indexes:

- Reference network: Solution[iRow, iCol] ← sign(Solution[iRow, iCol]), with sign(0) = 0.
- MRA and regression-based variants: MatrCc[iRow, iCol] ← sign(MatrCc[iRow, iCol]).
- In case a reduction to {0; 1} is necessary (edge existence only), the −1 in the above rules are replaced by 1.

To compute specificity and sensitivity when comparing with a reference network after discretization, we defined $T_{+}$ as the number of (iRow, iCol) such as MatrCc[iRow, iCol] = 1 and Solution[iRow, iCol] = 1. Same for $T_{-}$ and $T_{0}$. We defined $F_{+}$ as the number of (iRow, iCol) such as MatrCc[iRow, iCol] = 1 and Solution[iRow, iCol] ≠ 1. Same for${\;F}_{-}$ and $F_{0}$. We then defined P as the number of nonzero elements of Solution, *N* as the number of zero elements of Solution excluding the diagonal, and Se (sensitivity) = ($T_{+}$ + $T_{-}$)/*P*, Sp (specificity) = $T_{0}$*/N*. These definitions naturally extend the usual definitions of TP, TN, FP, FN, sensitivity, and specificity used by classification methods.

### 2.5 Generation of synthetic networks with FRANK

FRANK (Carré et al. 2017) was used requiring one eigenvalue of the TF matrix to reside on the unit circle to ensure reaching a steady state, dynamic mode, minimum sparsity 15% (meaning that 85% of the edges are null), and the other parameters were left at their default values. The output of FRANK provided us with matrices that we used as the exact, reference matrix of connectivity coefficients $r^{e}$ after setting the diagonal elements at −1 to comply with MRA formalism. To generate the input data for the inference methods, we had to compute the corresponding matrix R. From Equation (9), we obtain easily that $R=r^{-1}{\left(\text{diag}\left(r^{-1}\right)\right)}^{-1}\text{diag}(R)$, and diag(R) is known since we applied known perturbations to $X_{i}$ concentrations. For simulating full KOs we apply −100% to each $X_{i}$ and after Equation (6) all the diagonal elements of R are equal to −2. For a KD at −50% they are all equal to −2/3. Using the matrix $r^{e}$ from FRANK in these equations gives the corresponding R. Additive N(0,σ) noise was added to R with $\sigma =k\bar{X}$, k a factor adjusting the noise level (see Section 3) and $\bar{X}$ the average concentration of the genes.

### 2.6 Prior knowledge integration

Let us assume that we know some null edges of node $X_{i}$, i.e. a set $A_{i}=\left\{j\in \left\{1,\ldots ,N\right\};\;r_{ij}=0\right\}$.

Each index $j\in A_{i}$ cancels a column of the linear system associated with solving node $X_{i}$ connectivity coefficients. When the regressive approach is used, Equation (8) becomes:


(11) ΔXiXiqk=∑j=1, j≠ij∉ AiNrijΔXjXjqk.

Hence, the linear system corresponding to node $X_{i}\;$is reduced to $N-1-\text{card}(A_{i})$ parameters to estimate. The number of degrees of freedom of the model and its residual variance decrease accordingly.

## 3 Results and discussion

### 3.1 Impact of noise and perturbation intensities on MRA solutions

Conceptually, the derivation of MRA equations entails infinitesimal perturbations of the network modules (Kholodenko et al. 2002) such that differential calculus can be applied, notably the implicit function theorem that is a local result (Jimenez-Dominguez et al. 2021). In practice, infinitesimal perturbations are usually not feasible and not advisable because experimental data tend to be noisy. Nevertheless, Taylor’s development in Equation (4) shows a dependence of the error on the perturbation intensity (error term) underlying a potential loss of accuracy of MRA depending on the strengths of the variable nonlinear dependencies. Accordingly, we decided to study the relationship between accuracy, noise, and perturbation strengths on a small but representative system.

**Figure 1:**
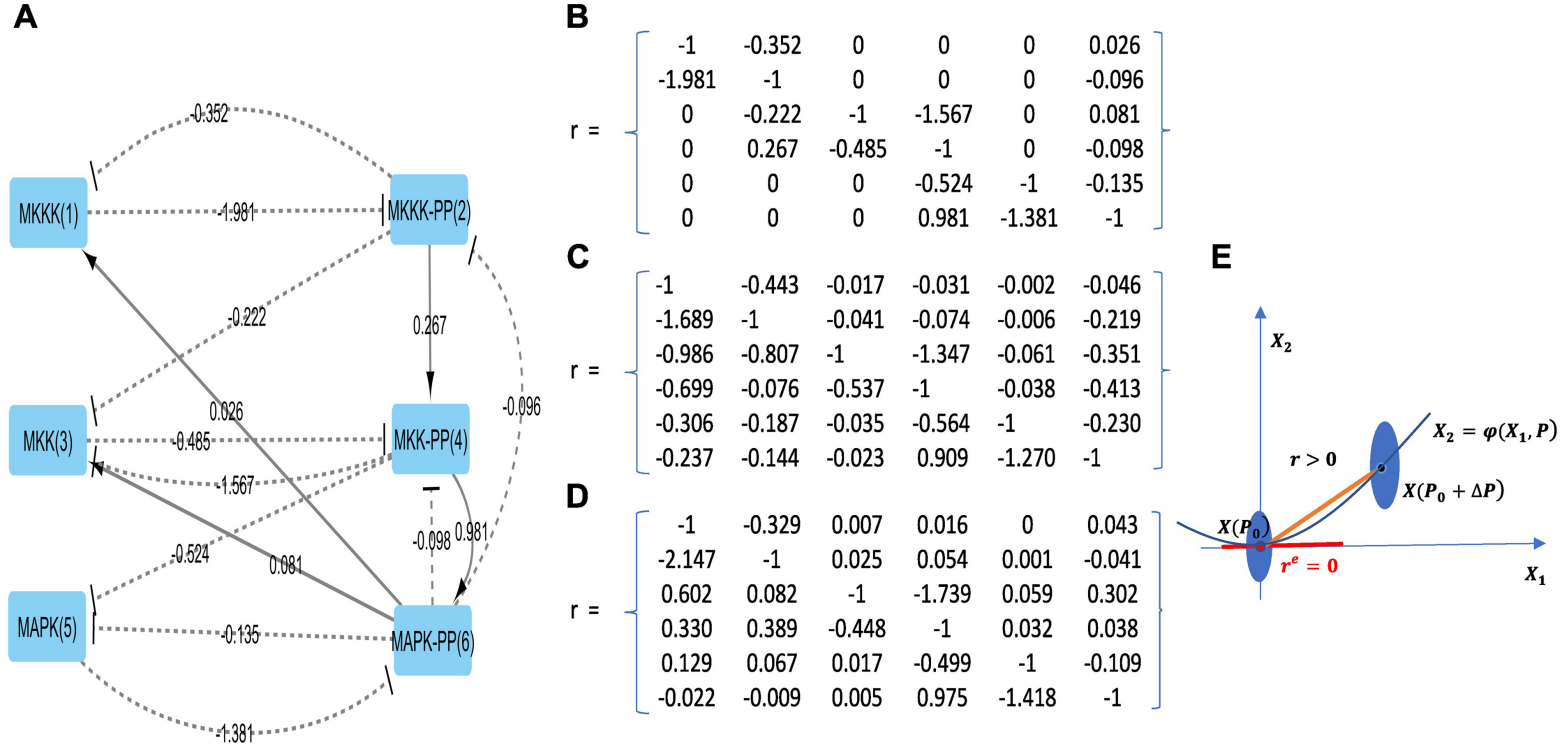
A six-node MAP kinase network. (A) Exact network topology with connectivity coefficients $r_{ij}^{e}$. Node names correspond to the different forms of kinases, and the numbers in brackets are their identifiers in further figures. A solid arrow means a direct activation, while a dashed arrow means an inhibition. (B) The matrix of exact $r_{ij}^{e}$. (C) Estimated $r_{ij}$ under −50% perturbation. (D) Estimated $r_{ij}$ under +50% perturbation. (E) Illustration of the impact of nonlinearity on the null edge problem. The exact connectivity coefficient $r^{e}=0$ (slope of the implicit function φ on $X(P_{0}$)) is approximated by the slope r>0 of the segment [$X\left(P_{0}\right),X\left(P_{0}+\Delta P\right)]$ in MRA (linear approximation)

We used the synthetic kinases network (MAP, MKK, and MKKK) introduced by Kholodenko et al. (2002) in MRA original paper. This system is comprised of six molecules (counting the phosphorylation states) and, unlike Kholodenko’s analysis that was limited to three modules, we considered the whole system with six modules, one *per* molecule (Fig. 1A). The dynamical equations are given in Supplementary Information. We solved them numerically using the R package ode and determined the time $t_{0}$ after which a steady state was reached (details in Supplementary Information). The exact connectivity coefficients $r_{ij}^{e}$ (Fig. 1B) were computed numerically.

We started with data devoid of noise and perturbations $q_{k}$ were applied to the six-speed constants of the system $v_{i}^{\text{max}}$, i∈{3,4,7,8,11,12} (see Supplementary Information) with ${\Delta P}_{k}$ equals to ±50%. Such perturbations could be induced by kinase inhibitors for instance. For each perturbation, a new steady state $X\left(P_{0}+{\Delta P}_{k}\right)$ was computed: the perturbation was applied to each node successively. For a + 50% perturbation level, every $v_{i}^{\text{max}}$ is successively multiplied by 1.5, while the others remain unchanged. Multiplication by 0.5 for a −50% perturbation. A first observation was that due to nonlinearity, symmetrical perturbations did not result in symmetrical differences compared to the basal state (Fig. 1C and D). In particular, the squared error was greater with −50% perturbations than with +50% perturbations. Moreover, we observed that connectivity coefficients that were zero in the basal state, were no more zero when estimated with standard MRA (Fig. 1B–D). Departure from zero was indeed substantial in such a small network, which is a limitation for identifying the topology of biological sparse networks (Fig. 1E).

**Figure 2:**
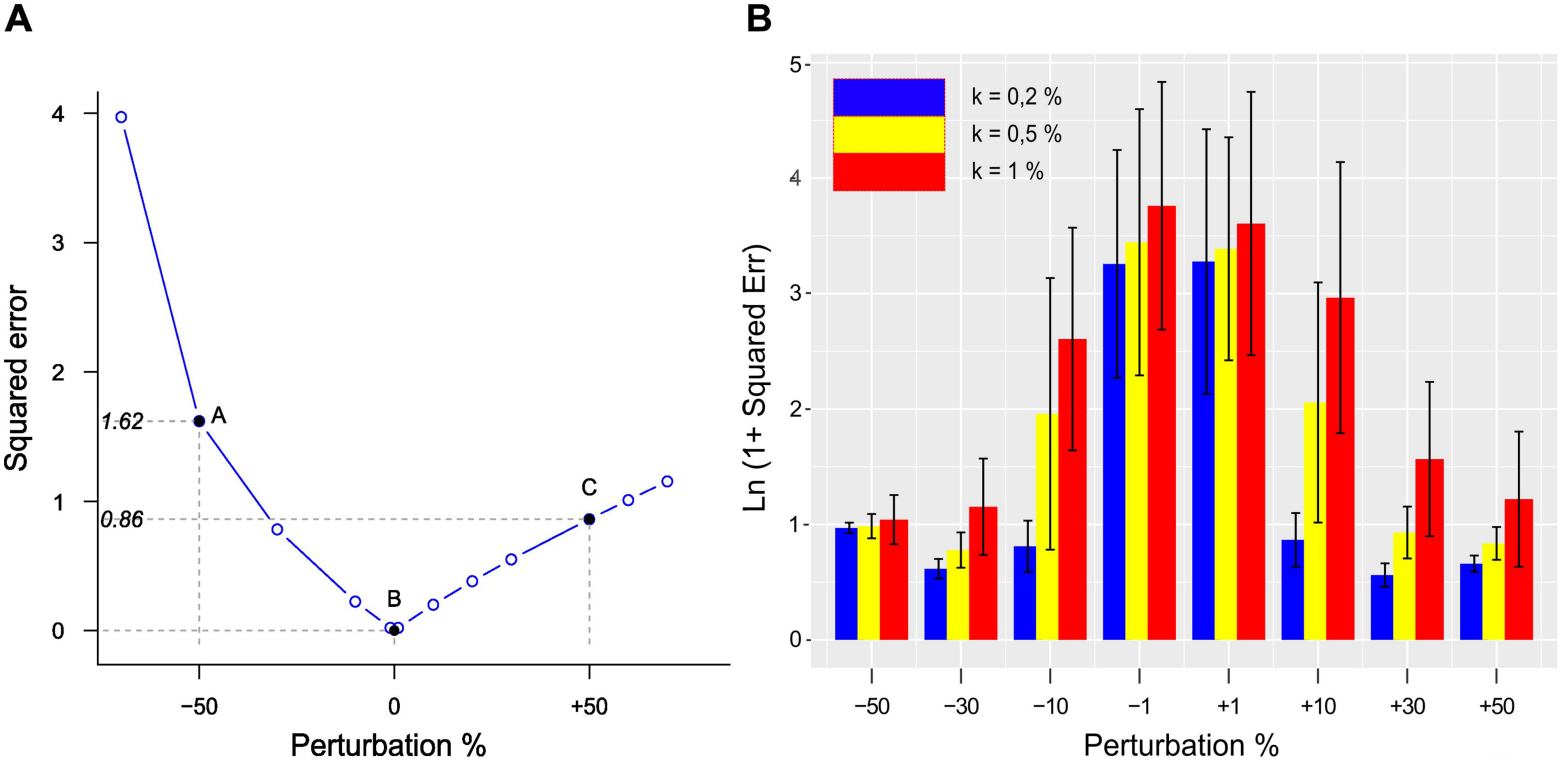
Impacts of perturbation and noise level for the six MAP kinase network. (A) The exact squared error $\sqrt{\sum_{i,j}{\left(r_{\mathrm{ij}}^{e}-r_{\mathrm{ij}}\right)}^{2}}$ as a function of the perturbation intensity in the absence of noise. We note that points A and C are not symmetrical with respect to B although symmetrical perturbations were applied. (B) The impact of perturbations at different intensities in the presence of Gaussian noise N(0,σ) with σ set at 0.1%, 0.5%, and 1% of the mean $\bar{X}$ of the molecule concentrations ($\sigma =k\bar{X}$). Black vertical lines indicate the error standard deviation. We note that the error is huge with small perturbations (−1% and +1%) when applied to noisier data. Stronger perturbations yield smaller errors provided the noise remains reasonable. Otherwise, errors grow rapidly as well (vertical axis in log)

Since the exact solution is available for the 6-node example, we can compute, for a given perturbation level, the exact squared error $\sqrt{\sum {\left(r_{ij}^{e}-r_{ij}\right)}^{2}\;}$. In Fig. 2A, we see that it is nonsymmetric as expected from the results above. To limit errors in $r_{ij}$ estimates, one could try using smaller perturbations, but as already mentioned, in the presence of noise, this approach might be inapplicable. To illustrate this phenomenon, three levels of Gaussian additive noise N(0,σ) were hence added to the $X_{i}$ with $\sigma =k\bar{X}$, where $\bar{X}$ was the average of all the theoretical concentrations at steady state ($\bar{X}$ = 84 for the example 6-node network), and k was set at 0.1%, 0.5%, or 1%. Note that with k = 1%, a substantial noise is added to low signals (k*=* 1% implies σ = 0. 84). The error in the presence of noise (Fig. 2B) behaved in a reverse fashion compared to what we observed without noise. This is because the noise error was larger than the nonlinearity error. With more noise, it is indeed necessary to increase perturbation intensities to obtain a sufficient signal-to-noise ratio, but this also drives us away from the exact solution in case the system is substantially nonlinear with respect to P (Equation (4)). In what follows, we used +50% perturbations as a reasonable compromise.

### 3.2 First comparison of methods

**Figure 3:**
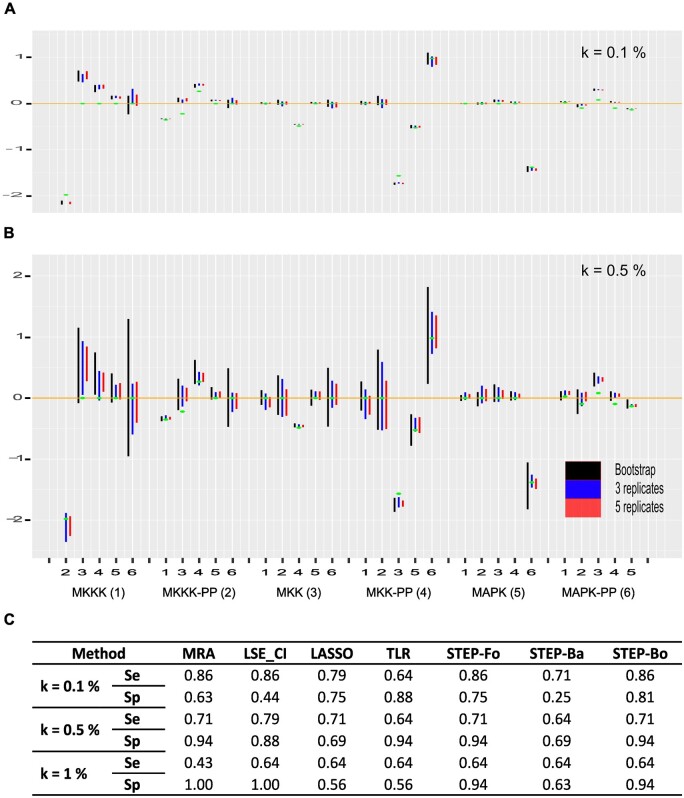
Inference performance for the six MAP kinase network. (A) Estimated connectivity coefficient $r_{ij}$ 95% CI for MRA standard resolution followed by a bootstrap, LSE with three or five replicates and Gaussian additive noise N(0,σ) with $\sigma =k\bar{X}$, $\bar{X}$ the average concentration, and k = 0.1%. Green dots represent the exact values. (B) Same as (A) but with k = 0.5%. The x-axes in (A) and (B) indicate the edge origins, e.g. MKKK, and the numbers the edge targets. (C) Performance estimations based on edge directionality and existence or absence at three noise levels. Se, sensitivity; Sp, specificity

MRA assesses network edges quantitatively, but measurement noise usually generates nonzero values for all the connectivity coefficients. Figure 3A and B illustrate this phenomenon for 6-node kinase network introduced above. For standard MRA, we estimated 95% CIs applying a bootstrap with 100 repetitions. A connectivity matrix r was obtained for each repetition of the bootstrap and CI boundaries were simply obtained as the 2.5th and 97.5th percentiles. We observed an already known relationship (Thomaseth et al. 2018): CI sizes increase with higher noise in the data, independent of the number of replicates. It is important to note that such CIs represent the consequence of the total variance (noise + nonlinearity), and by applying the standard MRA approach we are limited to such variance estimations *a posteriori*, e.g. via simulations such as the bootstrap. The independence on the number of replicates is an obvious limitation.

On the contrary, solving MRA with multilinear regression methods enables estimating the residual variance, i.e. the variance caused by the nonlinear contributions. Residual variance-based CIs is common practice when applying linear models to data. In Fig. 3A and B, we show such CIs for 3 and 5 replicates and noise levels at 0.1% and 0.5% of the average concentrations $\bar{X}$ for LSE regression. Residual variance-based CIs were indeed strongly reduced compared to standard MRA total variance-based CIs, and more replicates yielded smaller CIs. In a previous study limited to 3-module networks (Thomaseth et al. 2018), the idea of applying LSE has been already envisioned. Nonetheless, LSE was applied to each replicate separately thus leading to poor performance. In one case, the authors linked dual perturbations and reported encouraging performance, but they did not follow-up on this observation.

### 3.3 Determining edges existence in the 6-node network

In some applications, it is only necessary to infer the existence of node interactions and the sign of the interaction (induction or inhibition). To consider such applications, we reduced the exact connectivity coefficient $r_{ij}^{e}$ to −1, 0, or 1 by simply taking $\text{sign}(r_{ij}^{e})$. The output of MRA and MRA-regression methods were then also discretized to obtain values in {−1; 0; 1} (Section 2). Results are reported in Fig. 3C and Supplementary Table S1 (details of sensitivity and specificity computations are in Section 2 as well).

Already on this modest 6-node network, we see that MRA and MRA-regression performances depend on the noise level. Overall, the best sensitivity was achieved by STEP-Fo, LSE_CI, and LASSO (in this order), which outperformed MRA. TLR remained stable, regardless of the noise. Concerning specificity, the best results were obtained by MRA, STEP-Fo, and STEP-Bo. Upon noise increase, specificity was improved for MRA and LSE_CI because an increased noise inflated the CIs thus leading to the correct detection of every null edge (Supplementary Table S1).

### 3.4 Medium-size networks from DREAM 4 Challenge

**Figure 4:**
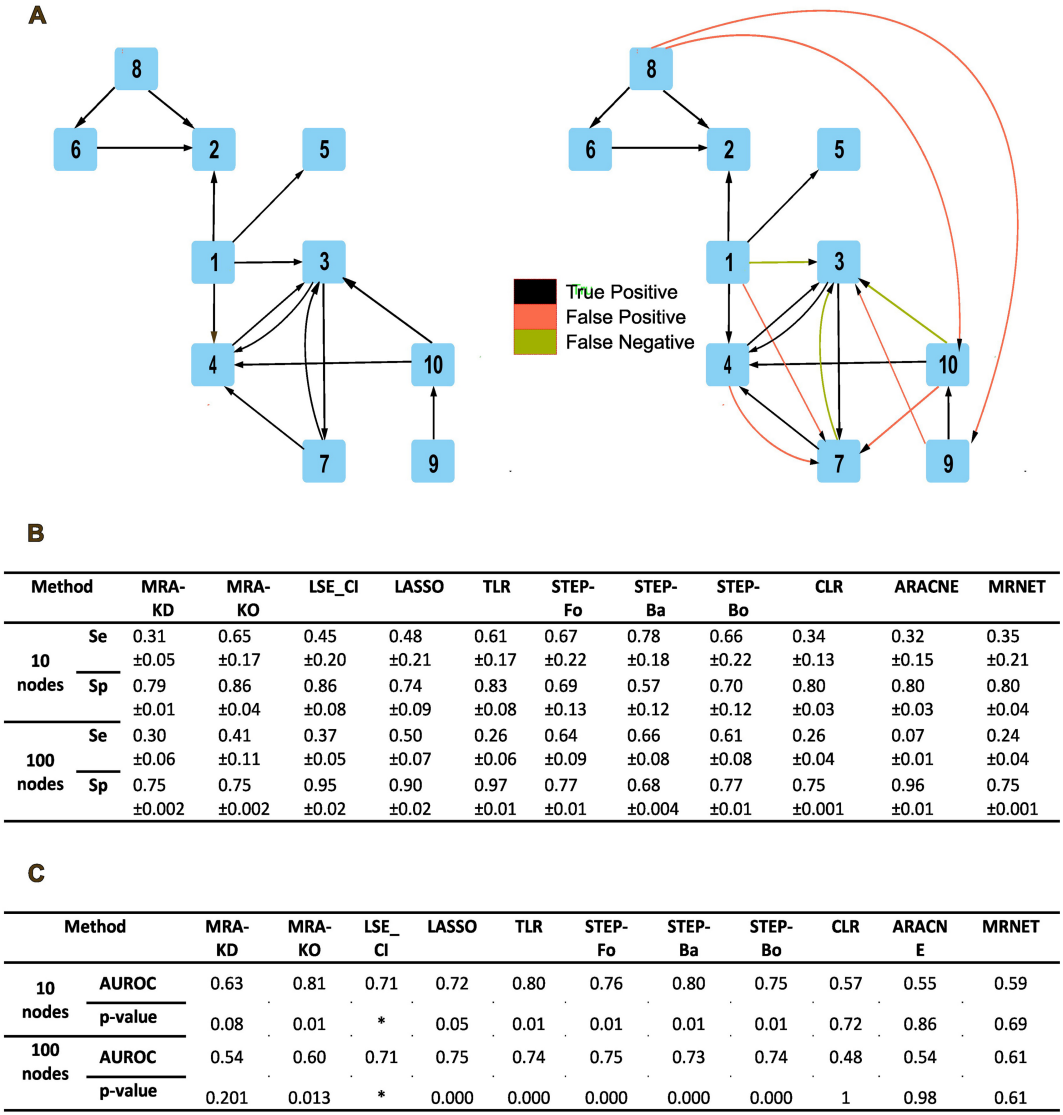
Dream 4 Challenge networks. (A) A 10-gene network topology (left) compared to TLR inference (right). (B) Average performance of the methods over the five 10- and five 100-gene networks. Se, sensitivity; Sp, specificity. (C) Average performance reported as area under the ROC curve (AUROC) and corresponding *P*-values over the five 10- and five 100-gene networks. *AUROC and *P*-values were computed using the library verification (function roc.area). For LSE_CI, it failed, and we estimated the AUROC by ourselves without *P*-value

The DREAM 4 Challenge released five 10-gene and five 100-gene networks of *Escherichia coli* meant to compare inference algorithm performance. Each gene was subjected to two independent perturbations series, knockdowns (KDs) and knockouts (KOs). All the gene expression levels $X_{i\;}$ were normalized and an unknown noise was added. The exact solutions were provided as binary edges, i.e. $r_{ij}^{e}\in \left\{0,1\right\}$. The networks were sparse with 80% of null edges on average. DREAM 4 data regarding the network dynamics before reaching a steady state were ignored. We evaluated the performance of our MRA-regression methods on the existence or nonexistence of the inferred edges, thus requiring discretization of the output real-valued $r_{i,j}$. In the case of MRA, it was not possible to combine KO and KD data in one analysis. We hence treated the two sets of perturbations separately. Regarding MRA output selection and discretization, we used x = 20% for 10-gene, and x = 25% for 100-gene networks (see Section 2). Figure 4A features the first 10-gene network compared with the result found by TLR, detailed results for each network are featured in Supplementary Table S2. Figure 4B reports the average performance over the five networks of each size in terms of sensitivity and specificity. To eliminate potential biases due to specific thresholds in the discretization, we also computed areas under the ROC curve (AUROC) featured in Fig. 4C.

On these larger networks, regressive approaches showed strong superiority over standard MRA (Fig. 4B and C). MRA-KD was close to random selection for 10- and 100-gene networks, whereas MRA-KO performed reasonably with 10-gene networks, but was also close to random with 100 genes (Supplementary Figs S1–S3). The simple regression method TLR delivered better performance, but was nonetheless sensitive to the network size. Regression methods adapted to sparse solutions (STEP-xx and LASSO) performed robustly and STEP-xx provided the best compromise overall. Comparing these methods with DREAM 4 Challenge ranking established in 2014, based on P-values concerning areas under the ROC curve (official criterion), we found that STEP-xx and TLR methods ranked 3rd for 10-gene networks. They respectively ranked 1st and 8th for 100-gene networks (STEP-Fo being the best of STEP-xx). See Supplementary Table S3 for detailed results and the exact score computation.

Naturally, these rankings do not take into account methods developed since DREAM 4. In particular, a variation of MRA called MLMSMRA (Klinger and Blüthgen 2018), using a likelihood estimation combined with a greedy hill-climbing model selection approach, ranked 3rd with 10-networks and KO perturbations, but 25th with KD. The authors did not report performance for 100-node networks. A Bayesian-modified version of MRA was proposed to better model sparse networks according to another ranking system defined by the authors themselves (Santra et al. 2013; Halasz et al. 2016; Klinger and Blüthgen 2018), i.e. achieving a performance comparable to MRA combined with STEP-xx.

### 3.5 Synthetic networks of sizes up to 1,000 nodes

We applied a realistic random biological network generator FRANK (Carré et al. 2017) to systematically explore performance on network sizes from 30 to 1,000 at two noise levels. We applied −50% (KD) and full KOs perturbations. We generated eight sets of networks with TF in (30, 60, 100, 200, 300, 500, 800, and 1000) and TA = 0. In FRANK terminology, TFs are genes regulating at least another gene (out-degree > 0), whereas TAs harbor out-degrees = 0. We also generated seven sets of networks, with TF = TA in (30, 50, 100, 150, 250, 400, and 500). The number of nodes (TF+TA) varied from 30 to 1000. For each set, we generated five independent networks with two different perturbations (KO and KD) and two noise levels. This resulted in $5\times \left(8+7\right)\times 2=150$ synthetic networks. FRANK sparsity parameter was set to have 85% of null edges.

**Figure 5:**
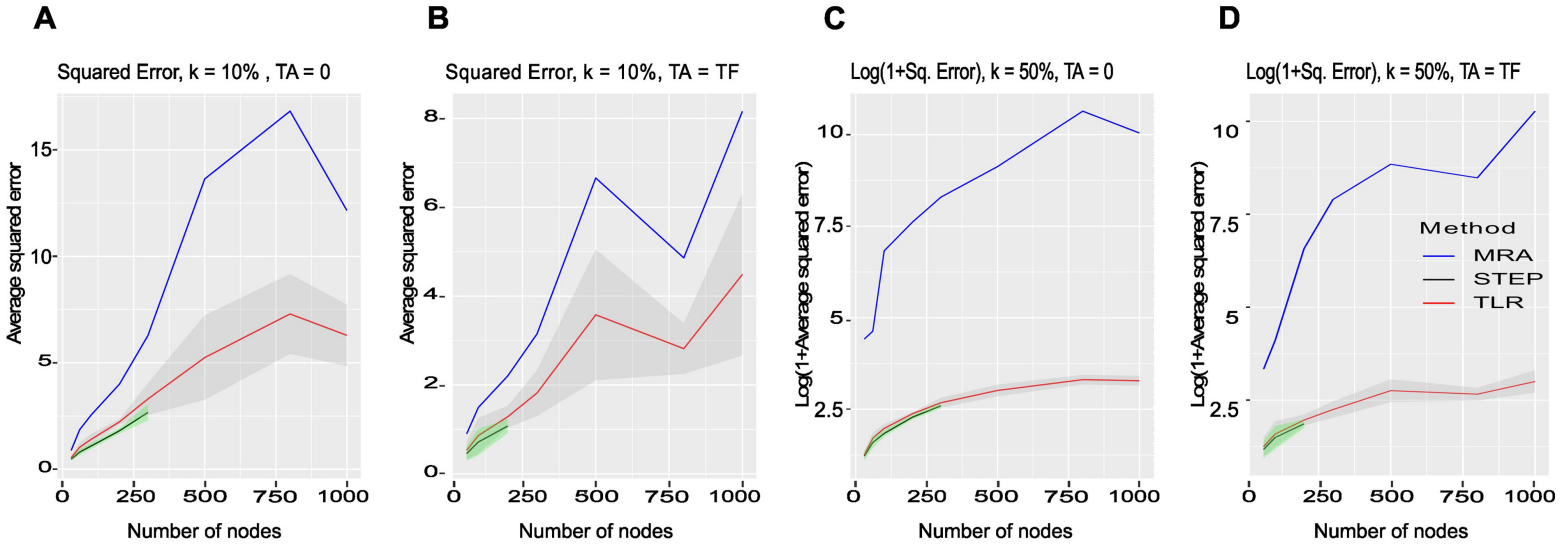
FRANK synthetic networks. (A) Squared error (SE) for MRA, TLR and STEP-Fo methods as a function of the number of nodes with additive Gaussian noise N(0,σ) and $\sigma =k\bar{X}$, $\bar{X}$ the average concentration, and k = 10%, number of nodes = TF, TA = 0. (B) Same with TA = TF, number of nodes = TA + TF. (C) Log (1 + SE) as a function of the number of nodes, k = 50%, number of nodes = TF, TA = 0. (D) Same with TA = TF, number of nodes = TA + TF. The ribbon around TLR and STEP-Fo curves features ± standard deviation of the error. For MRA, the error standard deviation was so large that it would encompass the whole plot. It was hence not represented. Since STEP-Fo was very compute-intensive, we limited its application to networks with TF ≤ 300 and TA = 0, or TF ≤ 100 and TA = TF

For each network and noise level, we computed the exact squared error $\sqrt{\sum {\left(r_{ij}^{e}-r_{ij}\right)}^{2}\;}$ for standard MRA and two regression-based methods that stood out in the DREAM 4 data, i.e. TLR and STEP-Fo. See Fig. 5 and Supplementary Table S4 for the results, which confirmed on this large collection of networks the significant improvement brought by the regression approach.

**Figure 6:**
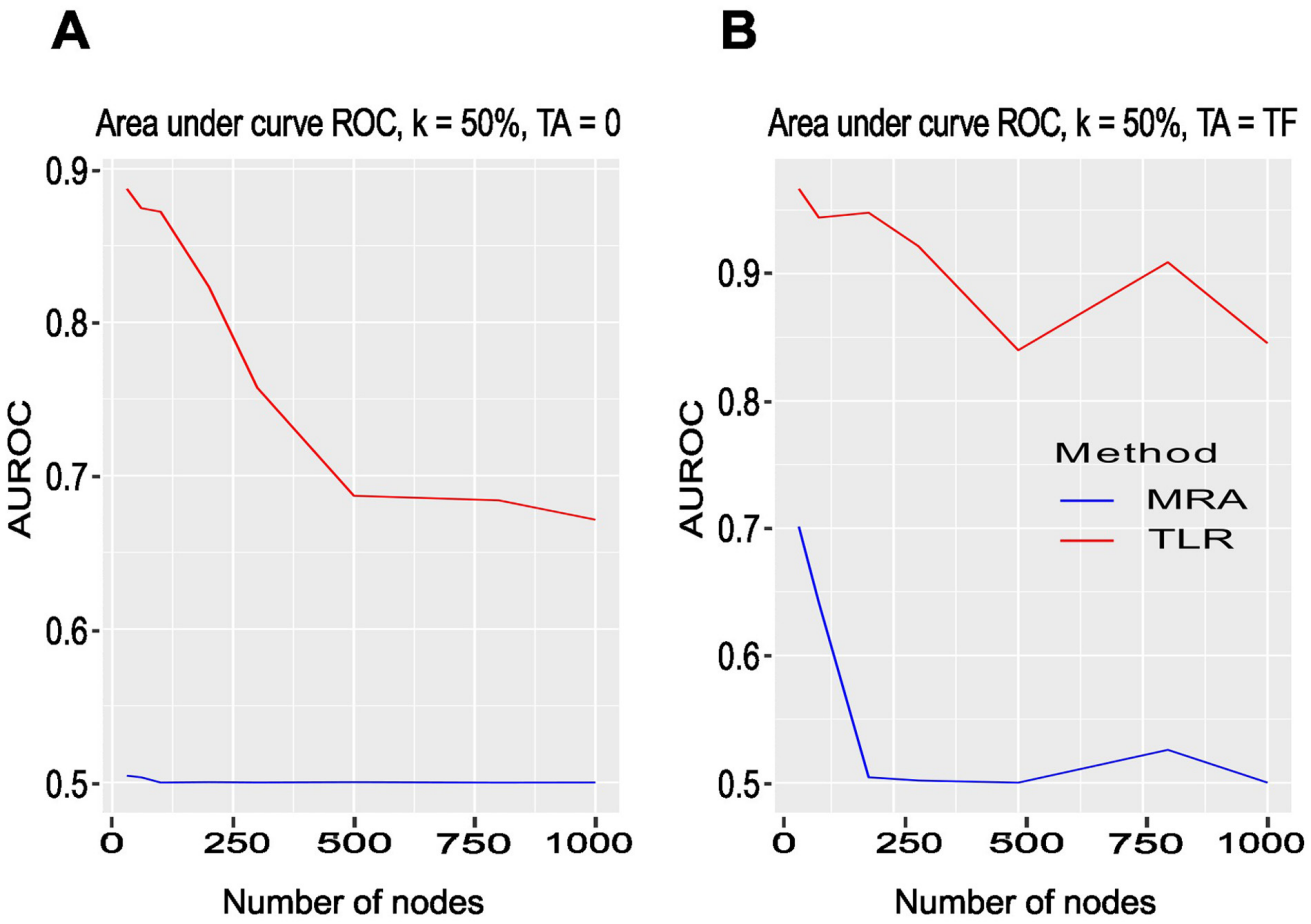
FRANK synthetic networks. (A) AUROC as a function of the number of nodes. Additive Gaussian noise N(0,σ) with $\sigma =k\bar{X}$, $\bar{X}$ the average concentration, and k = 50%. Number of nodes = TF, TA = 0. (B) Same with TA = TF, number of nodes = TA + TF. See Supplementary Table S5 for detailed results

To enable comparison with the network inference algorithms ARACNE, CLR, and MRNET, we next asked about the performance just detecting the existence of edges, i.e. both the reference connectivity coefficient $r_{ij}^{e}$ and their estimates $r_{ij}$ were discretized to {0; 1} (Section 2). Since AUROC computation is very time consuming, we limited performance assessment with AUROC to MRA and TLR (Fig. 6), which again showed the advantage of the regressive approach.

**Figure 7:**
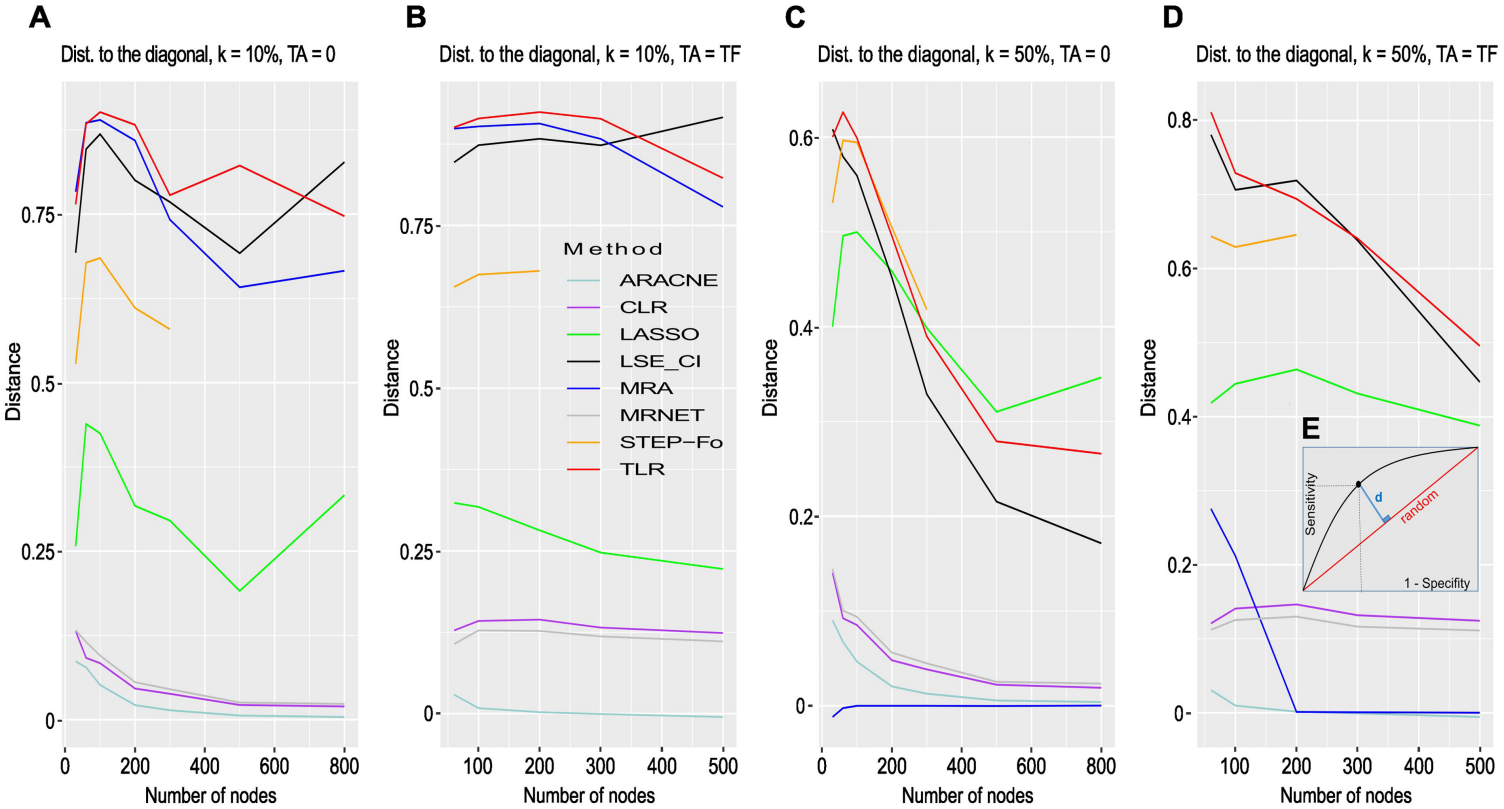
FRANK synthetic networks. (A) Distance to the diagonal as a function of the number of nodes with additive Gaussian noise N(0,σ) with $\sigma =k\bar{X}$, $\bar{X}$ the average concentration, and k = 10%, number of nodes = TF, TA = 0. (B) Same with TA = TF, number of nodes = TA + TF. (C) Distance to the diagonal as a function of the number of nodes, k = 50%, number of nodes = TF, TA = 0. (D) Same with TA = TF, number of nodes = TA + TF. (E) Distance of a point on a ROC curve to the diagonal. See Supplementary Table S6 for detailed results

In Fig. 7, we report the performance of all the methods by simply computing the distance of a single (sensitivity, 1 − specificity) point, obtained with default parameters, to the diagonal representing random selection. We clearly see that network inference methods do not perform well in comparison to MRA-based methods on perturbation data. We also see that standard MRA performs well with small noise but its performance drops dramatically as noise increases in line with previous results. The most robust methods are the simple TLR and STEP-Fo, but TLR outperforms STEP-Fo on this dataset, and it is much faster. LASSO performance is robust although seldom the best.

### 3.6 The use of *a priori* knowledge of the network topology

In practice, it is common to know about some interactions in a studied network. Regarding known edges, there is nothing particular to do since MRA formalism considers all the interactions as possible. On the other hand, by knowing impossible interactions, we can eliminate equations (see Section 2). This increases over determination of the linear system of the regression approach, which is expected to increase the accuracy of the estimates. For methods such as LASSO that require hyper-parameter adjustment through CV, this has the potential to improve the optimality of those hyper-parameters in addition.

**Figure 8:**
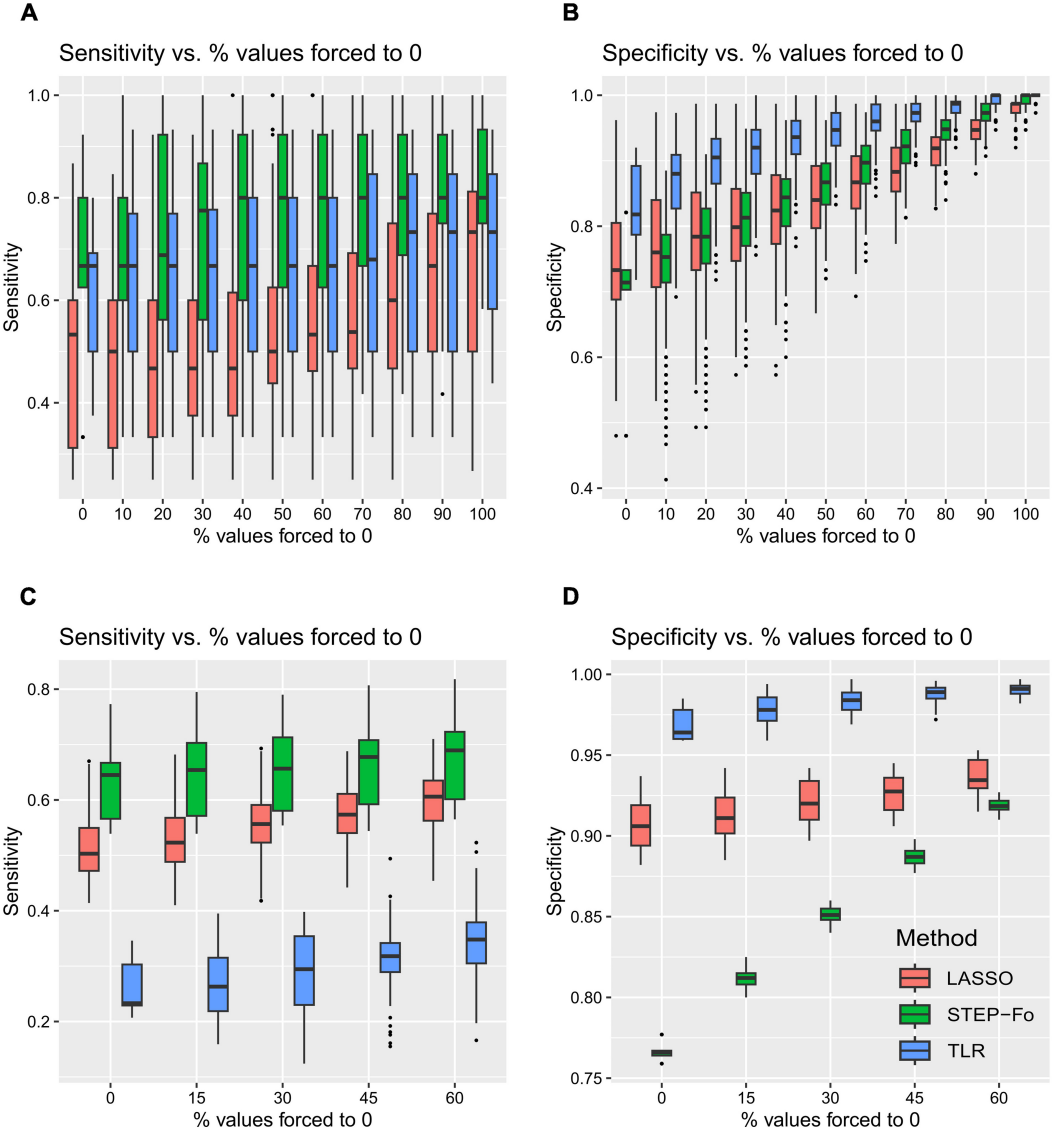
Existing knowledge impact on regression methods estimated on DREAM 4 Challenge networks. (A) 10-node networks, sensitivity. (B) 10-node networks, specificity. (C) 100-node networks, sensitivity. (D) 100-node networks, specificity

In the 10- and 100-gene networks of the DREAM 4 Challenge, we assumed different percentages of known null edges. The impact of this knowledge on sensibility and specificity for three of the studied regression methods (LASSO, STEP-Fo, and TLR) is featured in Fig. 8. Indeed, performance improved as a function of the percentage of known null edges. This improvement was stronger for specificity. We observed high variability of the impact on sensibility for 10-gene networks, results were more stable for the larger 100-gene networks.

In comparison with the binary approach proposed here, a Bayesian implementation of MRA has been proposed (Santra et al. 2013) that took a known pathway topology as a prior and showed improved accuracy over pure MRA. This Bayesian formulation was less radical than imposing null edges since an absent edge of the pathway could be eventually inferred *a posteriori*, provided that sufficient data evidence existed. On the other hand, our regression-based solution provides accurate CI estimates that hence enable pruning network edges based on model accuracy. One can imagine following with a reduced model, where pruned edges would be removed as above, to obtain better estimates on the other edges. This would be different from a Bayesian formulation obviously, though it would provide comparable practical value.

In the case of multilinear regression, the integration of the knowledge of null edges is done naturally (overfitting the system by deleting columns). In the Bayesian approach (Santra et al. 2013), the integration of this knowledge is also very natural (defining the prior probability of the Boolean variables associated with the existence of edges). In MLMSMRA (Klinger and Blüthgen 2018), the integration of prior knowledge was not explicitly discussed. Nonetheless, because their algorithm fills the connectivity matrix iteratively, it should be easy to force certain coefficients to remain at zero.

## 4 Conclusion

We have introduced a new method to solve MRA equations through multilinear regression. This formulation brings a number of advantages over the classical approach by providing a natural way to model data variability across experimental replicates, or even multiple perturbations at certain or all the modules. Better estimates of MRA connectivity coefficient variability can also be exploited to identify absent edges in a biological network more accurately. Moreover, these advantages were obtained by remaining within the MRA formalism that provides an elegant, physical interpretation of connectivity coefficients compared to purely regressive approaches.

While motivating the use of regression for MRA, we conducted an analysis of the relationship between data noise, perturbation intensity, and MRA result accuracy that has interest *per se*. In agreement with the local development in the Taylor series, MRA accuracy decreases with stronger perturbations as soon as the system is nonlinear. On the other hand, the presence of noise requires perturbations with a minimal intensity to obtain exploitable differences in the variables. Altogether, this means that a compromise must be found between noise levels and perturbation strengths. Depending on the biological system at hand, i.e. on the nonlinearities, this may lead to perturbations intensity no longer compatible with MRA linear approximation. In such a case, another modeling paradigm must be chosen. In the absence of strong nonlinearities, our work dramatically extended the domain of the application of MRA to much larger networks of sizes up to 1000. This is a 100-fold increase compared to MRA with standard linear algebra, which had difficulties going beyond 10-node networks in our experiments.

Finally, the proposed approach actually defines a family of MRA-derived methods with the multilinear regression algorithm as a free parameter. While LSE_CI for instance selects edges based on a direct exploitation of residual variance CIs, other algorithms perform model selection such as the simple although very efficient TLR, LASSO, or STEP-xx. Other regression methods that we have not tested might provide specific advantages depending on the dataset, or the specific interest or requirements of the researchers.

## Supplementary Material

btad166_Supplementary_Data

## Supplementary data

Financial Support: None declared.
