# Supplementary material for: Modular response analysis reformulated as a multilinear regression problem
Source: Bioinformatics. 2023 Apr 6;39(4):btad166. doi: 10.1093/bioinformatics/btad166 (PMC10097436; doi:10.1093/bioinformatics/btad166)
Supplement: btad166_Supplementary_Data [file btad166_supplementary_data.docx]

**SUPPLEMENTARY INFORMATION**

**Modular response analysis reformulated as a multilinear regression problem**

Jean-Pierre Borg^1,2,3^, Jacques Colinge^1,2,3,4^, Patrice Ravel^1,2,3,5^

^1^Institut de Recherche en Cancérologie de Montpellier, Inserm 1194, Montpellier, France

^2^Institut Régional du cancer Montpellier, Montpellier, France

^3^Université de Montpellier, Montpellier, France

^4^Faculté de Médecine, Montpellier, France

^5^Faculté de Pharmacie, Montpellier, France

**A 6-Node Model of MAP kinases**

This model was introduced in (Kholodenko *et al.*, 2002)

Concentrations and the Michaelis-Menten constants (K_ij_, i= 1, 3, 5, 7, 9, 11; j= 1, 2, 3; K_mp_; K_i_) as well as the catalytic rate constants (k_i_^cat^ , i= 1, 2, 5, 6, 9, 10) and the maximal enzyme rates (V_i_^max^, i= 3, 4, 7, 8, 11, 12) are provided by the authors in their Supplementary Material.

The kinetic equations and moiety conservations derived from the stoichiometry are the following:

d[MKKK-P]/dt = *v*_1_ - *v*_2_ + *v*_3_ – *v*_4_;

d[MKKK-PP]/dt = *v*_2_-*v*_3_;

d[MKK-P]/dt = *v*_5_ – *v*_6_ + *v*_7_ – *v*_8_;

d[MKK-PP]/dt = *v*_6_ – *v*_7_;

d[MAPK-P]/dt = *v*_9_ – *v*_10_ + *v*_11_ – *v*_12_;

d[MAPK-PP]/dt = *v*_10_ – *v*_11_;

[MKKK]_total_ = [MKKK] + [MKKK-P] + [MKKK-PP];

[MKK]_total_ = [MKK] + [MKK-P] + [MKK-PP];

[MAPK]_total_ = [MAPK] + [MAPK-P] + [MAPK-PP].

By inserting the conservation equations in the differential equations, we obtained a system of 6 differential equations that was integrated numerically with the ode R package. The values of the exact connectivity coefficients were computed based on the numerical approximations using the formula

$r_{ij}^{e}=-\left( \frac{X_{j}\frac{\partial f_{i}}{\partial X_{j}}}{X_{i}\frac{\partial f_{i}}{\partial X_{i}}} \right)$.

To determine a time $t_{0}=1000$[s] after which steady state was reached, we used R Deriv library to compute an expression for $\dot{X}(t)$ and found its roots with the library multiroot (one real-valued root was found only). We validated numerically that for $t>t_{0}$ the solution $X(t)$ remained constant.

**Software and hardware used for the development**

**Software**

R version 4.1.2 (2021-07-2) -- Platform: x86_64-pc-linux-gnu (64-bit)

RStudio 2022.07.2 Build 576

| **Package** | **Version** |
| --- | --- |
| deSolve | 1.34 |
| rootSolve | 1.8.2.3 |
| Deriv | 4.1.3 |
| forcats | 0.5.2 |
| ggplot2 | 3.4.0 |
| glmnet | 4.1.4 |
| data.table | 1.14.4 |
| dplyr | 1.0.10 |
| verification | 1.42 |
| pracma | 2.4.2 |
| minet | 3.52.0 |
| parallel | 4.1.2 |
| foreach | 1.5.2 |
| doParallel | 1.0.17 |

"minet" requires the package "Biocmanager" (v. 1.30.19). Then, BiocManager::install("minet").

**Hardware:**

PC Dell, core i7 - 14 cores, 2.4 GHz - RAM 64 GB.

**Our code:**

MRA3_BioInfo_2_Pub.R, available from GitHub: <https://github.com/J-P-Borg/BioInformatics>

**Supplementary Tables & Figures**

**k = 0,1%**

| **Method** | **T+** | **T-** | **T0** | **F+** | **F-** | **F0** | **Se** | **Sp** |
| --- | --- | --- | --- | --- | --- | --- | --- | --- |
| **MRA** | 4 | 8 | 10 | 8 | 0 | 0 | 0.86 | 0.63 |
| **LSE_CI** | 4 | 8 | 7 | 11 | 0 | 0 | 0.857 | 0.438 |
| **LASSO** | 4 | 7 | 12 | 4 | 1 | 2 | 0.786 | 0.75 |
| **TLR** | 3 | 6 | 14 | 2 | 0 | 5 | 0.643 | 0.875 |
| **STEP-Fo** | 4 | 8 | 12 | 4 | 1 | 1 | 0.857 | 0.75 |
| **STEP-Ba** | 3 | 7 | 4 | 11 | 2 | 3 | 0.714 | 0.25 |
| **STEP-Bo** | 4 | 8 | 13 | 4 | 0 | 1 | 0.857 | 0.812 |

**k = 0,5%**

| **Method** | **T+** | **T-** | **T0** | **F+** | **F-** | **F0** | **Se** | **Sp** |
| --- | --- | --- | --- | --- | --- | --- | --- | --- |
| **MRA** | 3 | 7 | 15 | 1 | 0 | 4 | 0.714 | 0.938 |
| **LSE_CI** | 4 | 7 | 14 | 3 | 0 | 2 | 0.786 | 0.875 |
| **LASSO** | 3 | 7 | 11 | 5 | 1 | 3 | 0.714 | 0.688 |
| **TLR** | 3 | 6 | 15 | 1 | 0 | 5 | 0.643 | 0.938 |
| **STEP-Fo** | 3 | 7 | 15 | 2 | 0 | 3 | 0.714 | 0.938 |
| **STEP-Ba** | 3 | 6 | 11 | 3 | 3 | 4 | 0.643 | 0.688 |
| **STEP-Bo** | 3 | 7 | 15 | 2 | 0 | 3 | 0.714 | 0.938 |

**k =1%**

| **Method** | **T+** | **T-** | **T0** | **F+** | **F-** | **F0** | **Se** | **Sp** |
| --- | --- | --- | --- | --- | --- | --- | --- | --- |
| **MRA** | 1 | 5 | 16 | 0 | 0 | 8 | 0.429 | 1.00 |
| **LSE_CI** | 2 | 7 | 16 | 0 | 0 | 5 | 0.643 | 1.00 |
| **LASSO** | 2 | 7 | 9 | 4 | 3 | 5 | 0.643 | 0.562 |
| **TLR** | 3 | 6 | 9 | 3 | 4 | 5 | 0.643 | 0.562 |
| **STEP-Fo** | 2 | 7 | 15 | 2 | 0 | 4 | 0.643 | 0.938 |
| **STEP-Ba** | 1 | 8 | 10 | 4 | 3 | 4 | 0.643 | 0.625 |
| **STEP-Bo** | 2 | 7 | 15 | 2 | 0 | 4 | 0.643 | 0.938 |

**Supplementary Table 1.** Confusion matrices for the 6 MAP kinase network and three levels of noise. T+ stands for true (correct) prediction of a positive coefficient, T- true prediction of a negative coefficient, T0 true prediction of a zero coefficient, F- is a predicted negative coefficient although its true value is positive or null, etc. Se stands for sensitivity and Sp for specificity. With $P$ the number of non-zero elements and $N$ the number of zero elements, then $Se= \frac{T_{+}+ T_{-}}{P}$ and $Sp= \frac{T_{0}}{N}$

| **inSilico_Size10_1** | **MRA-KD** | **MRA-KO** | **TLR** | **STEP-Fo** | **STEP-Ba** |
| --- | --- | --- | --- | --- | --- |
| **Se(nsitivity)** | 0.333 | 0.867 | 0.800 | 0.800 | 0.933 |
| **Sp(ecificity)** | 0.800 | 0.907 | 0.920 | 0.733 | 0.680 |
| **AUROC** | 0.602 | 0. 923 | 0. 909 | 0. 856 | 0.926 |

| **inSilico_Size10_2** | **MRA-KD** | **MRA-KO** | **TLR** | **STEP-Fo** | **STEP-Ba** |
| --- | --- | --- | --- | --- | --- |
| **Se(nsitivity)** | 0.375 | 0.562 | 0.375 | 0.625 | 0.750 |
| **Sp(ecificity)** | 0.811 | 0.851 | 0.892 | 0.703 | 0.568 |
| **AUROC** | 0.638 | 0.728 | 0.743 | 0.730 | 0.746 |

| **inSilico_Size10_3** | **MRA-KD** | **MRA-KO** | **TLR** | **STEP-Fo** | **STEP-Ba** |
| --- | --- | --- | --- | --- | --- |
| **Se(nsitivity)** | 0.267 | 0.733 | 0.667 | 0.667 | 0.800 |
| **Sp(ecificity)** | 0.787 | 0.880 | 0.787 | 0.480 | 0.360 |
| **AUROC** | 0.685 | 0.857 | 0.815 | 0.678 | 0.788 |

| **inSilico_Size10_4** | **MRA-KD** | **MRA-KO** | **TLR** | **STEP-Fo** | **STEP-Ba** |
| --- | --- | --- | --- | --- | --- |
| **Se(nsitivity)** | 0.308 | 0.692 | 0.692 | 0.923 | 0.923 |
| **Sp(ecificity)** | 0.792 | 0.857 | 0.818 | 0.714 | 0.623 |
| **AUROC** | 0.643 | 0.867 | 0.872 | 0.914 | 0.907 |

| **inSilico_Size10_5** | **MRA-KD** | **MRA-KO** | **TLR** | **STEP-Fo** | **STEP-Ba** |
| --- | --- | --- | --- | --- | --- |
| **Se(nsitivity)** | 0.250 | 0.417 | 0.500 | 0.333 | 0.500 |
| **Sp(ecificity)** | 0.782 | 0.808 | 0.718 | 0.821 | 0.615 |
| **AUROC** | 0.580 | 0.676 | 0.666 | 0.605 | 0.639 |

| **inSilico_Size100_1** | **MRA-KD** | **MRA-KO** | **TLR** | **STEP-Fo** | **STEP-Ba** |
| --- | --- | --- | --- | --- | --- |
| **Se(nsitivity)** | 0.341 | 0.585 | 0.233 | 0.773 | 0.773 |
| **Sp(ecificity)** | 0.749 | 0.753 | 0.985 | 0.767 | 0.677 |
| **AUROC** | 0.579 | 0.709 | 0.855 | 0.835 | 0.814 |

| **inSilico_Size100_2** | **MRA-KD** | **MRA-KO** | **TLR** | **STEP-Fo** | **STEP-Ba** |
| --- | --- | --- | --- | --- | --- |
| **Se(nsitivity)** | 0.373 | 0.378 | 0.229 | 0.566 | 0.614 |
| **Sp(ecificity)** | 0.751 | 0.751 | 0.978 | 0.764 | 0.683 |
| **AUROC** | 0.574 | 0.583 | 0.708 | 0.703 | 0.702 |

| **inSilico_Size100_3** | **MRA-KD** | **MRA-KO** | **TLR** | **STEP-Fo** | **STEP-Ba** |
| --- | --- | --- | --- | --- | --- |
| **Se(nsitivity)** | 0.267 | 0.379 | 0.303 | 0.667 | 0.697 |
| **Sp(ecificity)** | 0.748 | 0.750 | 0.960 | 0.777 | 0.680 |
| **AUROC** | 0.530 | 0.591 | 0.724 | 0.767 | 0.754 |

| **inSilico_Size100_4** | **MRA-KD** | **MRA-KO** | **TLR** | **STEP-Fo** | **STEP-Ba** |
| --- | --- | --- | --- | --- | --- |
| **Se(nsitivity)** | 0.294 | 0.422 | 0.346 | 0.645 | 0.664 |
| **Sp(ecificity)** | 0.748 | 0.751 | 0.959 | 0.759 | 0.673 |
| **AUROC** | 0.542 | 0.597 | 0.733 | 0.747 | 0.733 |

| **inSilico_Size100_5** | **MRA-KD** | **MRA-KO** | **TLR** | **STEP-Fo** | **STEP-Ba** |
| --- | --- | --- | --- | --- | --- |
| **Se(nsitivity)** | 0.233 | 0.295 | 0.207 | 0.539 | 0.565 |
| **Sp(ecificity)** | 0.747 | 0.748 | 0.964 | 0.766 | 0.676 |
| **AUROC** | 0.472 | 0.531 | 0.687 | 0.680 | 0.666 |

**Supplementary Table 2.** Performance achieved for each network of the Dream Challenge 4 with MRA, TLR and STEP.

**Computation of the methods' ranking in Dream Challenge 4**

We applied the exact rules of the DREAM 4 Challenge, which are the following:

- We compute the AUROC (R method "roc.area", with the parameters "exact solution" and "computed values").
- We compute the corresponding p-value, using the method wilcox.test (Mann-Whitney U). This value is delivered by "roc.area" : $p.value. We call the p-values corresponding to networks **_1, **_2… **_5: PV1, PV2... PV5.
- From these values for one network size (10 or 100), we compute the AUROC SCORE:

- LOG10((PV1*PV2*PV3*PV4*PV5)^0,2).

- At last, we insert the values of our 4 methods in the available DREAM 4 list and our ranks, see Table S3 below.

Hereafter the scores of all the methods submitted to the DREAM 4 Challenge with our scores inserted in red.

| **InSilicoSize10** | | | | | | | |
| --- | --- | --- | --- | --- | --- | --- | --- |
| **Rank** | **Competitor** | **PV1** | **PV2** | **PV3** | **PV4** | **PV5** | **AUROC Score** |
| 1 | amalia | 4,45E-009 | 5,37E-006 | 9,53E-010 | 9,62E-008 | 6,51E-007 | 7,17 |
| 2 | X | 6,27E-09 | 6,99E-05 | 2,57E-07 | 2,01E-06 | 1,38E-04 | 5,70 |
| 3 | STEP-Ba | 9,84E-010 | 0,000322424 | 0,000127684 | 6,15E-008 | 0,03522547 | 5,01 |
| 4 | STEP-Bo | 6,21E-008 | 0,000168223 | 0,01437743 | 4,84E-009 | 0,04007165 | 4,51 |
| 5 | STEP-Fo | 6,21E-008 | 0,000229966 | 0,009315064 | 6,88E-009 | 0,04007165 | 4,49 |
| 6 | TLR | 2,43E-007 | 0,001099545 | 5,38E-005 | 8,28E-006 | 0,03202285 | 4,08 |
| 7 | X | 1,27E-05 | 2,31E-02 | 3,75E-06 | 6,59E-07 | 5,75E-03 | 4,08 |
| 8 | X | 1,39E-06 | 2,50E-02 | 5,03E-06 | 5,23E-06 | 2,50E-02 | 3,93 |
| 9 | X | 1,39E-05 | 1,78E-03 | 1,47E-04 | 1,90E-04 | 2,29E-04 | 3,76 |
| 10 | X | 7,92E-06 | 1,09E-02 | 9,31E-06 | 1,90E-04 | 8,60E-03 | 3,58 |
| 11 | X | 7,60E-05 | 5,81E-07 | 2,23E-02 | 1,06E-03 | 2,42E-03 | 3,52 |
| 12 | X | 4,21E-06 | 2,14E-01 | 3,98E-05 | 1,13E-04 | 9,07E-04 | 3,49 |
| 13 | X | 5,65E-04 | 9,50E-02 | 2,91E-05 | 4,66E-07 | 1,08E-02 | 3,42 |
| 14 | X | 5,24E-06 | 1,05E-01 | 1,78E-05 | 8,69E-06 | 3,02E-01 | 3,32 |
| 15 | X | 8,45E-04 | 7,90E-02 | 1,94E-04 | 1,70E-06 | 6,81E-03 | 3,16 |
| 16 | X | 5,66E-06 | 5,51E-01 | 6,38E-05 | 4,99E-05 | 9,59E-02 | 3,00 |
| 17 | X | 1,10E-05 | 3,44E-02 | 2,61E-04 | 3,86E-04 | 3,30E-02 | 2,98 |
| 18 | X | 8,12E-05 | 1,19E-01 | 2,19E-05 | 7,26E-04 | 2,17E-02 | 2,90 |
| 19 | X | 2,27E-04 | 4,45E-03 | 1,68E-01 | 9,02E-03 | 2,42E-05 | 2,69 |
| 20 | X | 9,43E-03 | 1,37E-02 | 3,65E-03 | 3,09E-06 | 2,74E-01 | 2,48 |
| 21 | X | 3,71E-04 | 6,59E-01 | 3,25E-06 | 1,28E-03 | 1,00E+00 | 2,40 |
| 22 | X | 5,87E-03 | 9,40E-03 | 9,29E-03 | 9,88E-03 | 9,48E-03 | 2,06 |
| 23 | X | 5,21E-02 | 2,05E-03 | 1,39E-03 | 1,53E-02 | 2,65E-02 | 2,04 |
| 24 | X | 2,22E-04 | 2,94E-01 | 1,10E-02 | 1,34E-02 | 3,25E-02 | 1,90 |
| 25 | X | 5,55E-02 | 1,27E-01 | 2,40E-03 | 2,05E-03 | 6,93E-02 | 1,72 |
| 26 | X | 3,28E-02 | 7,16E-03 | 4,63E-01 | 3,14E-02 | 3,04E-02 | 1,40 |
| 27 | X | 2,90E-02 | 7,65E-02 | 1,16E-02 | 7,76E-02 | 6,23E-02 | 1,38 |
| 28 | X | 5,93E-02 | 3,96E-01 | 1,50E-01 | 1,25E-02 | 1,52E-02 | 1,23 |
| 29 | X | 2,43E-02 | 1,52E-01 | 2,46E-01 | 1,37E-01 | 8,06E-03 | 1,20 |
| 30 | X | 9,52E-02 | 9,70E-02 | 2,90E-02 | 2,01E-02 | 3,02E-01 | 1,16 |
| 31 | X | 9,25E-01 | 6,46E-01 | 1,61E-01 | 4,66E-03 | 8,03E-02 | 0,89 |
| 32 | X | 4,43E-01 | 7,07E-01 | 2,79E-01 | 2,01E-02 | 8,79E-02 | 0,76 |
| 33 | X | 5,92E-01 | 2,38E-01 | 4,50E-01 | 7,02E-01 | 2,47E-02 | 0,59 |

| **InSilicoSize100** | | | | | | | |
| --- | --- | --- | --- | --- | --- | --- | --- |
| **Rank** | **Competitor** | **PV1** | **PV2** | **PV3** | **PV4** | **PV5** | **AUROC Score** |
| 1 | STEP-Fo | 1,26E-092 | 2,96E-048 | 1,17E-067 | 8,46E-060 | 4,55E-031 | 59,15 |
| 2 | STEP-Bo | 5,97E-085 | 5,88E-048 | 4,30E-060 | 1,49E-059 | 3,87E-029 | 55,61 |
| 3 | STEP-Ba | 2,60E-066 | 8,29E-040 | 6,89E-049 | 5,23E-044 | 8,98E-022 | 43,43 |
| 4 | X | 1.916e-41 | 3.123e-43 | 1.063e-50 | 1.334e-42 | 1.818e-30 | 40,96 |
| 5 | ALF | 6.214e-41 | 4.325e-45 | 3.187e-48 | 6.503e-43 | 5.070e-27 | 40,11 |
| 6 | X | 3.920e-40 | 6.988e-44 | 3.639e-47 | 2.051e-43 | 1.015e-27 | 39,74 |
| 7 | X | 8.215e-40 | 5.861e-39 | 1.194e-48 | 2.559e-44 | 3.031e-28 | 39,27 |
| 8 | X | 3.340e-39 | 2.325e-41 | 3.977e-43 | 2.051e-43 | 3.931e-25 | 37,72 |
| 9 | X | 1.879e-34 | 8.767e-38 | 2.836e-51 | 6.714e-40 | 7.634e-27 | 37,32 |
| 10 | TLR | 3,00E-059 | 1,97E-029 | 2,95E-027 | 2,07E-031 | 3,05E-019 | 32,59 |
| 11 | X | 1.408e-33 | 6.720e-32 | 1.107e-35 | 3.641e-36 | 1.765e-20 | 30,83 |
| 12 | X | 7.663e-32 | 3.743e-30 | 3.114e-34 | 2.563e-30 | 2.169e-16 | 27,86 |
| 13 | X | 3.243e-23 | 2.988e-26 | 1.966e-38 | 3.339e-28 | 2.313e-16 | 25,77 |
| 14 | X | 4.148e-18 | 2.876e-36 | 1.989e-19 | 2.816e-23 | 2.071e-30 | 24,77 |
| 15 | X | 7.828e-24 | 7.700e-20 | 1.350e-19 | 9.384e-22 | 4.271e-12 | 18,70 |
| 16 | X | 8.938e-26 | 1.014e-16 | 2.038e-18 | 2.444e-21 | 2.489e-07 | 17,19 |
| 17 | X | 8.204e-19 | 2.503e-19 | 3.711e-20 | 3.368e-17 | 3.169e-11 | 16,62 |
| 18 | X | 9.553e-13 | 1.077e-08 | 9.149e-25 | 6.264e-18 | 1.539e-13 | 14,81 |
| 19 | X | 7.490e-12 | 2.529e-05 | 1.196e-05 | 7.758e-10 | 6.921e-06 | 6,98 |
| 20 | X | 1.270e-04 | 1.644e-06 | 1.443e-07 | 1.277e-06 | 3.619e-06 | 5,57 |
| 21 | X | 4.151e-03 | 2.946e-02 | 2.007e-01 | 2.237e-05 | 8.349e-05 | 2,67 |
| 22 | X | 5.143e-01 | 6.298e-01 | 1.000e+00 | 7.644e-01 | 9.977e-01 | 0,12 |
| 23 | X | 9.358e-01 | 9.578e-01 | 9.763e-01 | 9.686e-01 | 9.581e-01 | 0,02 |

**Supplementary Table 3.** Ranking of the regression methods within Dream Challenge 4

*Squared Error. TA = 0. k=0.1*

| **TF** | | **30** | **60** | **100** | **200** | **300** | **500** | **800** | **1000** |
| --- | --- | --- | --- | --- | --- | --- | --- | --- | --- |
| **MRA** | avg | 0.881 | 1.857 | 2.541 | 3.997 | 6.277 | 13.638 | 16.820 | 12.152 |
|  | sd | 0.135 | 0.379 | 0.575 | 0.243 | 1.858 | 11.903 | 7.828 | 3.825 |
| **TLR** | avg | 0.538 | 1.035 | 1.401 | 2.226 | 3.300 | 5.247 | 7.293 | 6.289 |
|  | sd | 0.095 | 0.211 | 0.269 | 0.124 | 0.753 | 1.983 | 1.875 | 1.445 |
| **STEP-Fo** | avg | 0.474 | 0.809 | 1.089 | 1.808 | 2.668 |  |  |  |
|  | sd | 0.072 | 0.147 | 0.139 | 0.134 | 0.381 |  |  |  |

*Squared Error. TA = 0. k=0.5*

| **TF** | | **30** | **60** | **100** | **200** | **300** | **500** | **800** | **1000** |
| --- | --- | --- | --- | --- | --- | --- | --- | --- | --- |
| **MRA** | avg | 99.867 | 110.610 | 2024.581 | 2355.514 | 7584.28 | 22368.29 | 107370.0 | 24453.428 |
|  | sd | 60.433 | 44.988 | 2766.248 | 1297;611 | 11013.94 | 32448.55 | 163317.4 | 8996.847 |
| **TLR** | avg | 2.516 | 4.599 | 6.272 | 9.812 | 13.679 | 19.611 | 26.596 | 25.761 |
|  | sd | 0.455 | 0.810 | 0.936 | 0.465 | 2.046 | 3.410 | 3.600 | 3.696 |
| **STEP-Fo** | avg | 2.414 | 3.973 | 5.327 | 8.854 | 12.423 |  |  |  |
|  | sd | 0.452 | 0.698 | 0.591 | 0.625 | 1.041 |  |  |  |

*Squared Error. TA = TF. k=0.1*

| **TF** | | **30** | **50** | **100** | **150** | **250** | **400** | **500** |
| --- | --- | --- | --- | --- | --- | --- | --- | --- |
| **MRA** | avg | 0.900 | 1.495 | 2.204 | 3.152 | 6.658 | 4.860 | 8.161 |
|  | sd | 0.367 | 0.720 | 0.419 | 0.978 | 3.137 | 1.051 | 3.748 |
| **TLR** | avg | 0.530 | 0.863 | 1.285 | 1.820 | 3.579 | 2.818 | 4.489 |
|  | sd | 0.211 | 0.400 | 0.244 | 0.519 | 1.471 | 0.570 | 1.826 |
| **STEP-Fo** | avg | 0.455 | 0.718 | 1.076 |  |  |  |  |
|  | sd | 0.160 | 0.297 | 0.157 |  |  |  |  |

*Squared Error. TA = TF. k=0.5*

| **TF** | | **30** | **50** | **100** | **150** | **250** | **400** | **500** |
| --- | --- | --- | --- | --- | --- | --- | --- | --- |
| **MRA** | avg | 148.176 | 168.048 | 1528.957 | 3650.716 | 7394.361 | 18910.75 | 46331.19 |
|  | sd | 268.531 | 172.519 | 1565.253 | 2584.277 | 2638.484 | 25602.78 | 41535.82 |
| **TLR** | avg | 2.583 | 4.135 | 6.198 | 8.652 | 15.342 | 13.483 | 19.949 |
|  | sd | 0.941 | 1.783 | 1.087 | 2.225 | 4.905 | 2.485 | 6.525 |
| **STEP-Fo** | avg | 2.305 | 3.652 | 5.486 |  |  |  |  |
|  | sd | 0.782 | 1.486 | 0.748 |  |  |  |  |

**Supplementary Table 4.** Squared Error averaged on the five networks of each set defined by FRANK generator, for MRA, TLR and STEP-Fo methods. Results for very large TF were not computed for STEP-Fo method because of the huge computation time required.

*AUROC. TA = 0. k=0.1*

| **TF** | **30** | **60** | **100** | **200** | **300** | **500** | **800** | **1000** |
| --- | --- | --- | --- | --- | --- | --- | --- | --- |
| **MRA** | 0.99 | 0.99 | 0.99 | 0.99 | 0.98 | 0.91 | 0.94 | 0.93 |
| **TLR** | 0.99 | 1.00 | 1.00 | 0.99 | 0.99 | 0.98 | 0.99 | 0.98 |

*AUROC. TA = 0. k=0.5*

| **TF** | **30** | **60** | **100** | **200** | **300** | **500** | **800** | **1000** |
| --- | --- | --- | --- | --- | --- | --- | --- | --- |
| **MRA** | 0.50 | 0.50 | 0.50 | 0.50 | 0.50 | 0.50 | 0.50 | 0.50 |
| **TLR** | 0.89 | 0.87 | 0.87 | 0.82 | 0.76 | 0.69 | 0.68 | 0.67 |

*AUROC. TA = TF. k=0.1*

| **TF** | **30** | **50** | **100** | **150** | **250** | **400** | **500** |
| --- | --- | --- | --- | --- | --- | --- | --- |
| **MRA** | 1.00 | 1.00 | 1.00 | 0.99 | 0.99 | 0.99 | 0.99 |
| **TLR** | 1.00 | 1.00 | 1.00 | 1.00 | 1.00 | 1.00 | 1.00 |

*AUROC. TA = TF. k=0.5*

| **TF** | **30** | **50** | **100** | **150** | **250** | **400** | **500** |
| --- | --- | --- | --- | --- | --- | --- | --- |
| **MRA** | 0.70 | 0.64 | 0.50 | 0.50 | 0.50 | 0.53 | 0.50 |
| **TLR** | 0.97 | 0.94 | 0.95 | 0.92 | 0.84 | 0.91 | 0.85 |

**Supplementary Table 5.** Area Under ROC curves **(**"AUROC"), averaged on the five networks of each set defined by FRANK generator, for MRA and TLR methods.

*Se / Sp. TA = 0. k=0.1*

| **TF** | **30** | **60** | **100** | **200** | **300** | **500** | **800** | **1000** |
| --- | --- | --- | --- | --- | --- | --- | --- | --- |
| **MRA** | 1  0.79 | 0.95  0.93 | 0.93  0.96 | 0.94  0.92 | 0.85  0.90 | 0.76  0.88 | 0.73  0.94 | 0.88  0.79 |
| **LSE_CI** | 1  0.69 | 1  0.85 | 1  0.87 | 1  0.80 | 1  0.77 | 1  0.69 | 1  0.83 |  |
| **LASSO** | 1  0.26 | 1  0.44 | 1  0.43 | 1  0.32 | 1  0.30 | 1  0.19 | 1  0.33 |  |
| **TLR** | 1  0.76 | 0.97  0.92 | 0.96  0.94 | 0.97  0.91 | 0.89  0.89 | 0.96  0.87 | 0.80  0.94 | 0.98  0.76 |
| **STEP-Fo** | 1  0.53 | 1  0.68 | 1  0.68 | 1  0.61 | 1  0.58 |  |  |  |
| **CLR** | 0.58  0.55 | 0.55  0.54 | 0.54  0.55 | 0.48  0.57 | 0.46  0.58 | 0.48  0.54 | 0.49  0.53 | 0.43  0.58 |
| **MRA_CLR** | 0.55  0.73 | 0.44  0.79 | 0.28  0.84 | 0.52  0.49 | 0.66  0.36 | 0.63  0.38 | 0.81  0.19 | 1.00  0.00 |
| **ARACNE** | 0.18  0.91 | 0.12  0.96 | 0.08  0.97 | 0.04  0.98 | 0.03  0.99 | 0.01  0.99 | 0.01  1.00 | 0.01  1.00 |
| **MRNET** | 0.59  0.54 | 0.56  0.56 | 0.53  0.56 | 0.48  0.58 | 0.45  0.59 | 0.47  0.55 | 0.48  0.55 |  |

*Se / Sp. TA = TF. k=0.1*

| **TF** | **30** | **50** | **100** | **150** | **250** | **400** | **500** |
| --- | --- | --- | --- | --- | --- | --- | --- |
| **MRA** | 0.98  0.92 | 0.94  0.96 | 0.93  0.97 | 0.91  0.98 | 0.79  0.99 | 0.93  0.96 | 0.83  0.98 |
| **LSE_CI** | 1  0.85 | 1  0.87 | 1  0.88 | 1  0.87 | 1  0.92 |  |  |
| **LASSO** | 0.99  0.34 | 0.98  0.34 | 1  0.28 | 1  0.25 | 1  0.22 |  |  |
| **TLR** | 0.98  0.92 | 0.96  0.96 | 0.96  0.97 | 0.94  0.98 | 0.83  1.00 | 0.98  0.96 | 0.88  0.97 |
| **STEP-Fo** | 1.00  0.66 | 1.00  0.67 | 1.00  0.68 |  |  |  |  |
| **CLR** | 0.52  0.61 | 0.54  0.60 | 0.54  0.61 | 0.52  0.62 | 0.53  0.60 | 0.53  0.62 | 0.52  0.62 |
| **MRA_CLR** | 0.38  0.73 | 0.20  0.93 | 0.52  0.45 | 0.60  0.37 | 0.74  0.24 | 1.00  0.00 | 1.00  0.00 |
| **ARACNE** | 0.10  0.93 | 0.06  0.95 | 0.03  0.97 | 0.02  0.98 | 0.01  0.98 | 0.01  0.99 | 0.01  0.99 |
| **MRNET** | 0.51  0.60 | 0.52  0.61 | 0.51  0.61 | 0.50  0.62 | 0.50  0.61 |  |  |

*Se / Sp. TA = 0. k=0.5*

| **TF** | **30** | **60** | **100** | **200** | **300** | **500** | **800** | **1000** |
| --- | --- | --- | --- | --- | --- | --- | --- | --- |
| **MRA** | 0.01  0.98 | 0.01  0.98 | 0.01  0.99 | 0.00  1.00 | 0.00  1.00 | 0.00  1.00 | 0.00  1.00 | 0.00  1.00 |
| **LSE_CI** | 0.76  0.84 | 0.66  0.92 | 0.64  0.92 | 0.53  0.92 | 0.41  0.92 | 0.29  0.92 | 0.24  0.93 |  |
| **LASSO** | 0.86  0.54 | 0.86  0.63 | 0.88  0.62 | 0.85  0.61 | 0.78  0.62 | 0.67  0.64 | 0.68  0.67 |  |
| **TLR** | 0.88  0.72 | 0.78  0.85 | 0.72  0.88 | 0.63  0.87 | 0.56  0.84 | 0.47  0.81 | 0.41  0.86 | 0.44  0.81 |
| **STEP-Fo** | 0.94  0.59 | 0.89  0.71 | 0.90  0.69 | 0.84  0.67 | 0.76  0.66 |  |  |  |
| **CLR** | 0.59  0.55 | 0.56  0.53 | 0.54  0.54 | 0.49  0.56 | 0.46  0.58 | 0.49  0.54 | 0.49  0.53 | 0.44  0.58 |
| **MRA_CLR** | 0.26  0.75 | 0.22  0.79 | 0.21  0.79 | 0.22  0.79 | 0.21  0.79 | 0.14  0.86 | 0.14  0.86 | 0.13  0.87 |
| **ARACNE** | 0.18  0.91 | 0.11  0.95 | 0.07  0.97 | 0.04  0.98 | 0.03  0.99 | 0.01  0.99 | 0.01  1.00 | 0.01  1.00 |
| **MRNET** | 0.61  0.54 | 0.56  0.54 | 0.54  0.55 | 0.49  0.57 | 0.46  0.59 | 0.48  0.54 | 0.48  0.54 |  |

*Se / Sp. TA = TF. k=0.5*

| **TF** | **30** | **50** | **100** | **150** | **250** | **400** | **500** |
| --- | --- | --- | --- | --- | --- | --- | --- |
| **MRA** | 0.34  0.94 | 0.26  0.95 | 0.04  1.00 | 0.00  1.00 | 0.00  1.00 | 0.01  1.00 | 0.00  1.00 |
| **LSE_CI** | 0.90  0.88 | 0.81  0.90 | 0.80  0.92 | 0.72  0.92 | 0.51  0.94 |  |  |
| **LASSO** | 0.90  0.52 | 0.91  0.54 | 0.92  0.55 | 0.87  0.56 | 0.77  0.62 |  |  |
| **TLR** | 0.88  0.93 | 0.78  0.95 | 0.73  0.97 | 0.68  0.96 | 0.55  0.95 | 0.64  0.95 | 0.55  0.94 |
| **STEP-Fo** | 0.98  0.66 | 0.95  0.68 | 0.96  0.69 |  |  |  |  |
| **CLR** | 0.51  0.61 | 0.54  0.60 | 0.54  0.61 | 0.52  0.62 | 0.53  0.60 | 0.53  0.62 | 0.52  0.62 |
| **MRA_CLR** | 0.37  0.70 | 0.25  0.67 | 0.17  0.85 | 0.15  0.88 | 0.15  0.87 | 0.16  0.87 | 0.14  0.88 |
| **ARACNE** | 0.10  0.93 | 0.06  0.95 | 0.03  0.97 | 0.02  0.98 | 0.01  0.98 | 0.01  0.99 | 0.01  0.99 |
| **MRNET** | 0.51  0.60 | 0.52  0.61 | 0.52  0.62 | 0.49  0.62 | 0.50  0.61 |  |  |

**Supplementary Table 6.** Sensitivity and specificity averaged over the five networks of each network size, noise level, and topology (TA=TF or TA=0) generated by FRANK. Some results were not computed because of the excessive compute time required (empty cells).


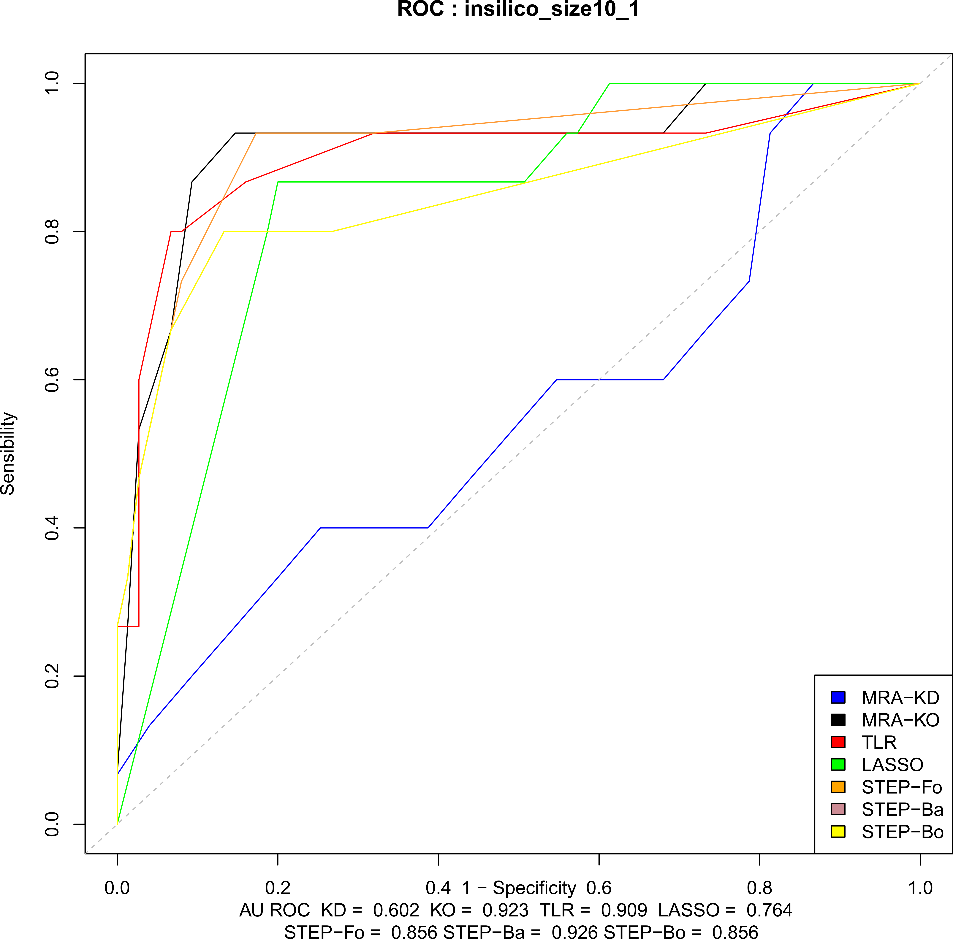


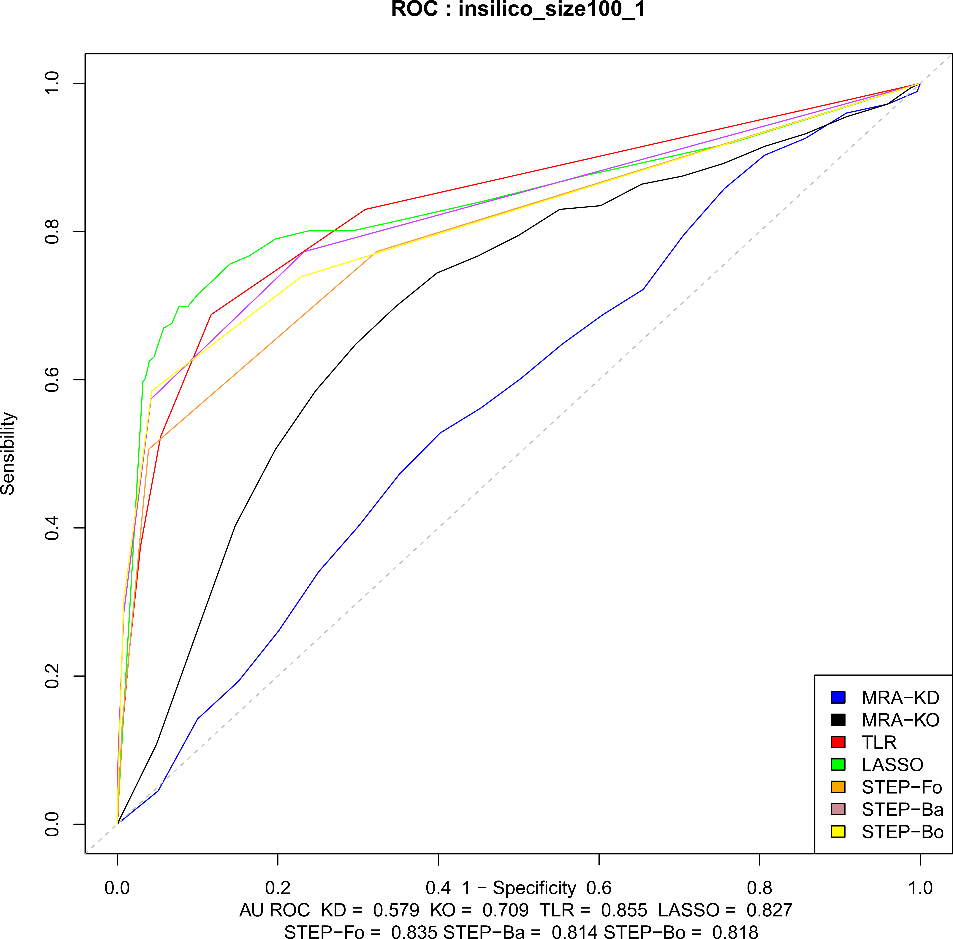


**Supplementary Figure 1.** ROC curves for DREAM 4 Challenge.

| **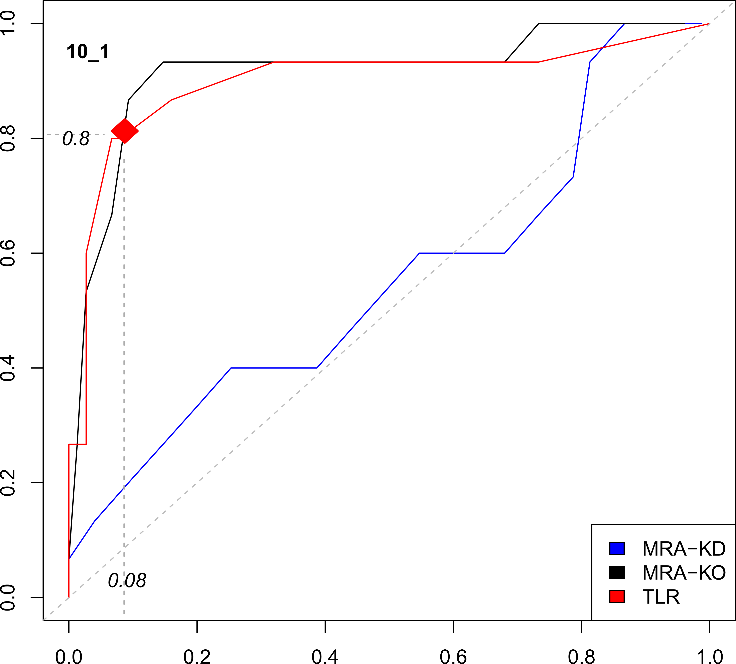** | **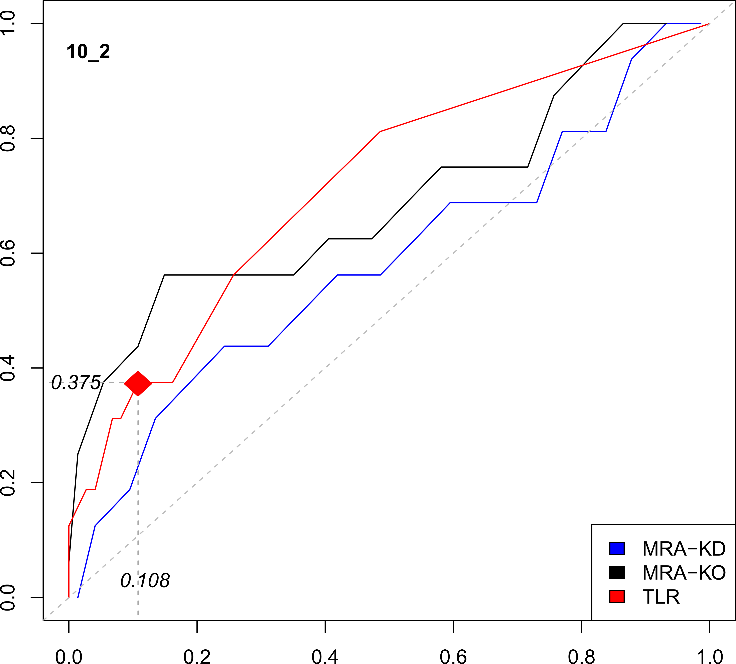** |  |
| --- | --- | --- |
| **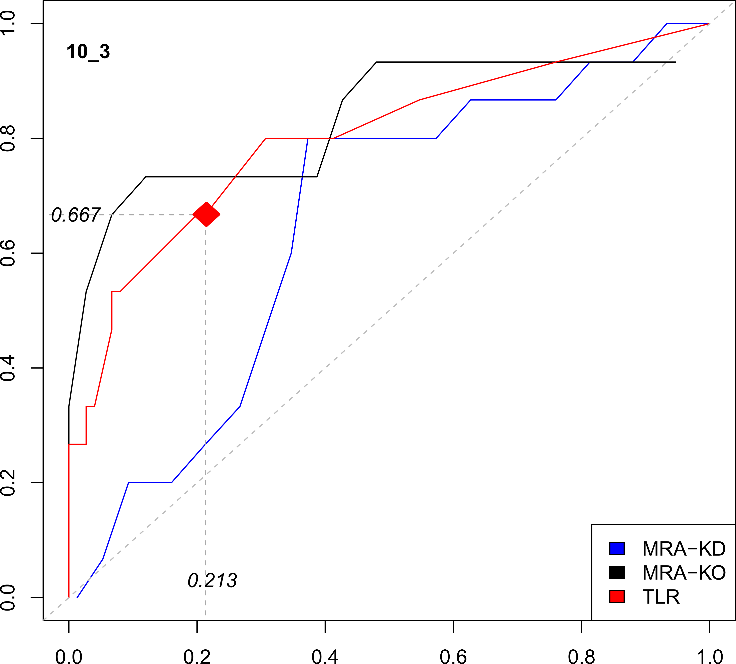** | **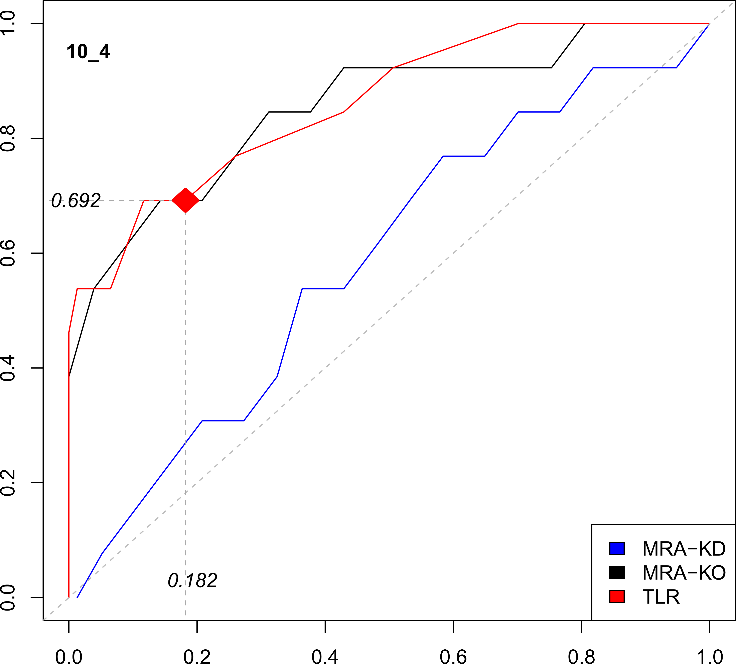** |  |
| **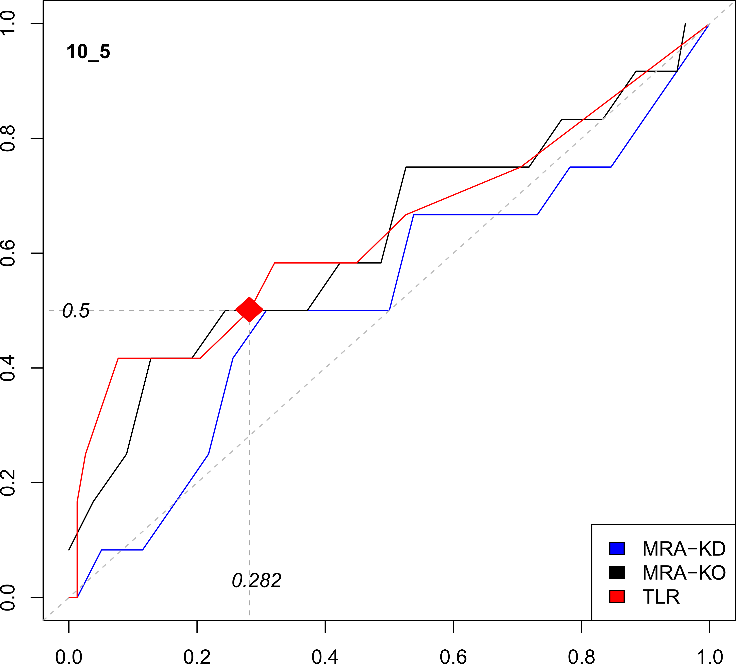** | \| **AUC** \| **10-1** \| **10-2** \| **10-3** \| **10-4** \| **10-5** \| **Mean** \| **sd** \| \| --- \| --- \| --- \| --- \| --- \| --- \| --- \| --- \| \| **MRA-KD** \| 0.60 \| 0.64 \| 0.68 \| 0.64 \| 0.58 \| 0.63 \| 0.04 \| \| **MRA-KO** \| 0.92 \| 0.73 \| 0.86 \| 0.87 \| 0.68 \| 0.81 \| 0.10 \| \| **TLR** \| 0.91 \| 0.74 \| 0.81 \| 0.87 \| 0.67 \| 0.80 \| 0.10 \| \| **STEP-Fo** \| 0.86 \| 0.73 \| 0.68 \| 0.91 \| 0.61 \| 0.76 \| 0.13 \| \| **STEP-Ba** \| 0.93 \| 0.75 \| 0.79 \| 0.91 \| 0.64 \| 0.80 \| 0.12 \| \| **STEP-Bo** \| 0.86 \| 0.73 \| 0.66 \| 0.91 \| 0.61 \| 0.75 \| 0.13 \| |  |

**Supplementary Figure 2.** Representative ROC curves and all the areas under the ROC curve for each DREAM 4 Challenge 10-node network. X-axis = 1-Sp(ecificity). Y-axis = Se(nsitivity). The dot corresponds to the default threshold of TLR (0.25 * max (|$r_{ij}$|).

| 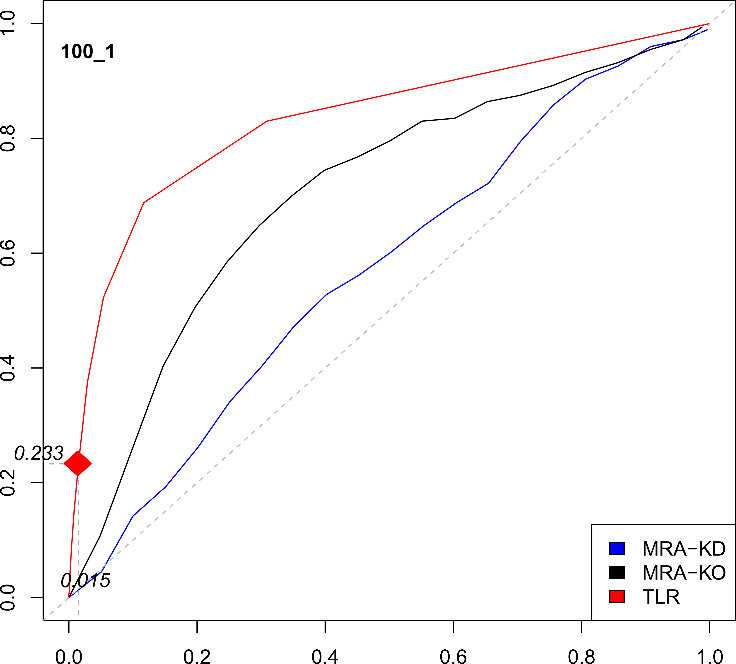 | 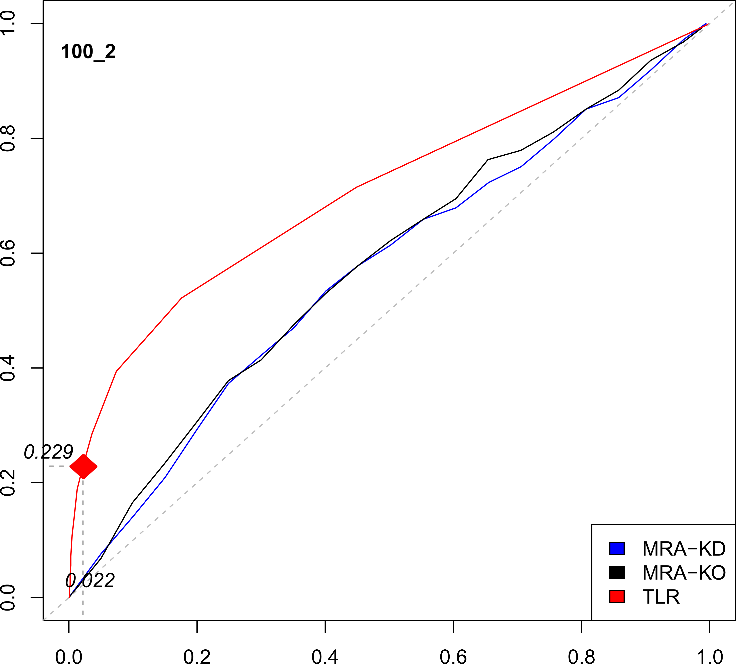 |
| --- | --- |
| 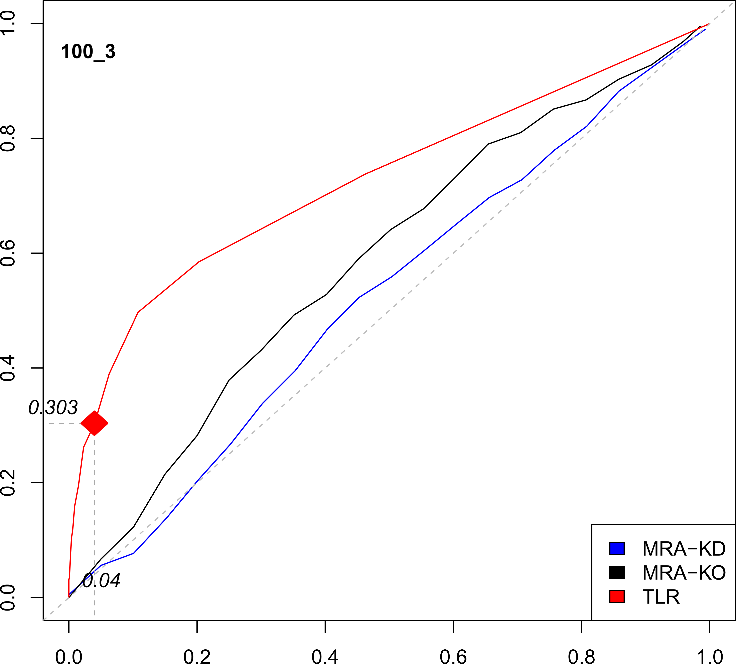 | 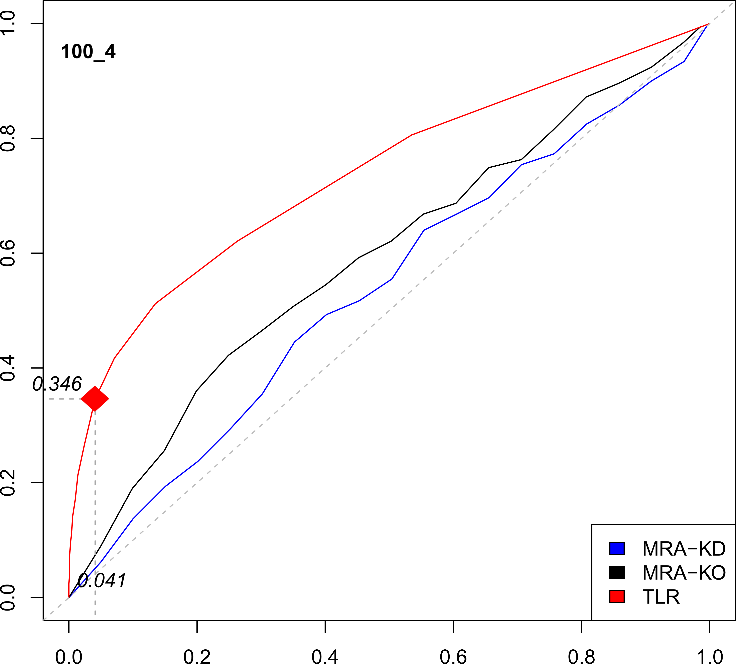 |
| 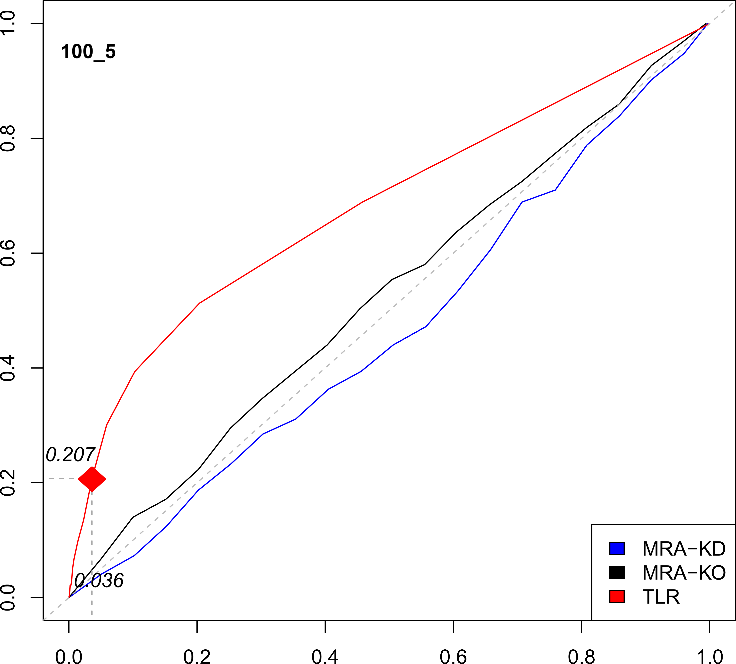 | \| **AUC** \| **100-1** \| **100-2** \| **100-3** \| **100-4** \| **100-5** \| **Mean** \| **sd** \| \| --- \| --- \| --- \| --- \| --- \| --- \| --- \| --- \| \| **MRA-KD** \| 0.58 \| 0.57 \| 0.53 \| 0.54 \| 0.47 \| 0.54 \| 0.04 \| \| **MRA-KO** \| 0.71 \| 0.58 \| 0.59 \| 0.6 \| 0.53 \| 0.6 \| 0.06 \| \| **TLR** \| 0.86 \| 0.71 \| 0.72 \| 0.73 \| 0.69 \| 0.74 \| 0.07 \| \| **STEP-Fo** \| 0.84 \| 0.7 \| 0.77 \| 0.75 \| 0.68 \| 0.75 \| 0.06 \| \| **STEP-Ba** \| 0.81 \| 0.7 \| 0.75 \| 0.73 \| 0.67 \| 0.73 \| 0.06 \| \| **STEP-Bo** \| 0.82 \| 0.7 \| 0.75 \| 0.74 \| 0.67 \| 0.74 \| 0.06 \| |

**Supplementary Figure 3.** Representative ROC curves and all the areas under the ROC curve for each DREAM 4 Challenge 100-node network. X-axis = 1-Sp(ecificity). Y-axis = Se(nsitivity). The dot corresponds to the default threshold of TLR (0.25 * max (|$r_{ij}$|).
